# Ethnic disparities in COVID-19 outcomes: a multinational cohort study of 20 million individuals from England and Canada

**DOI:** 10.1186/s12889-023-15223-8

**Published:** 2023-02-27

**Authors:** Francesco Zaccardi, Pui San Tan, Baiju R. Shah, Karl Everett, Ash Kieran Clift, Martina Patone, Defne Saatci, Carol Coupland, Simon J. Griffin, Kamlesh Khunti, Hajira Dambha-Miller, Julia Hippisley-Cox

**Affiliations:** 1 Leicester Real World Evidence Unit, Leicester Diabetes Centre, University of Leicester, Leicester, England; 2 Nuffield Department of Primary Care Health Sciences, University of Oxford, Oxford, England; 3 Department of Medicine, University of Toronto; Division of Endocrinology, Sunnybrook Health Sciences Centre, Institute for Clinical Evaluative Sciences, Toronto, Canada; 4 Cancer Research UK Oxford Centre, Department of Oncology, University of Oxford, Oxford, England; 5 Division of Primary Care, School of Medicine, University of Nottingham, Nottingham, England; 6 Primary Care Unit, School of Clinical Medicine, University of Cambridge, Cambridge, England; 7 MRC Epidemiology Unit, School of Clinical Medicine, University of Cambridge, Cambridge, England; 8 Primary Care Research Centre, University of Southampton, Southampton, England

**Keywords:** Ethnicity, COVID-19, Mortality, Hospitalisation, UK, Canada, Inequalities

## Abstract

**Background:**

Heterogeneous studies have demonstrated ethnic inequalities in the risk of SARS-CoV-2 infection and adverse COVID-19 outcomes. This study evaluates the association between ethnicity and COVID-19 outcomes in two large population-based cohorts from England and Canada and investigates potential explanatory factors for ethnic patterning of severe outcomes.

**Methods:**

We identified adults aged 18 to 99 years in the QResearch primary care (England) and Ontario (Canada) healthcare administrative population-based datasets (start of follow-up: 24th and 25th Jan 2020 in England and Canada, respectively; end of follow-up: 31st Oct and 30th Sept 2020, respectively). We harmonised the definitions and the design of two cohorts to investigate associations between ethnicity and COVID-19-related death, hospitalisation, and intensive care (ICU) admission, adjusted for confounders, and combined the estimates obtained from survival analyses. We calculated the ‘percentage of excess risk mediated’ by these risk factors in the QResearch cohort.

**Results:**

There were 9.83 million adults in the QResearch cohort (11,597 deaths; 21,917 hospitalisations; 2932 ICU admissions) and 10.27 million adults in the Ontario cohort (951 deaths; 5132 hospitalisations; 1191 ICU admissions). Compared to the general population, pooled random-effects estimates showed that South Asian ethnicity was associated with an increased risk of COVID-19 death (hazard ratio: 1.63, 95% CI: 1.09-2.44), hospitalisation (1.53; 1.32-1.76), and ICU admission (1.67; 1.23-2.28). Associations with ethnic groups were consistent across levels of deprivation. In QResearch, sociodemographic, lifestyle, and clinical factors accounted for 42.9% (South Asian) and 39.4% (Black) of the excess risk of COVID-19 death.

**Conclusion:**

International population-level analyses demonstrate clear ethnic inequalities in COVID-19 risks. Policymakers should be cognisant of the increased risks in some ethnic populations and design equitable health policy as the pandemic continues.

**Supplementary Information:**

The online version contains supplementary material available at 10.1186/s12889-023-15223-8.

## Introduction

There have been almost 450 million SARS-CoV-2 infections and 6 million deaths (as of March 2022) worldwide since the novel coronavirus disease 2019 (COVID-19) pandemic emerged [1]. Several studies have demonstrated ethnic inequalities in the risk of infection and adverse outcomes, which has generated substantial concern [2–5]. In the United Kingdom (UK), compared to White individuals, men from all backgrounds other than Chinese, and women from any ethnic group other than Bangladeshi, Chinese or mixed ethnicity, had an increased risk of COVID-19 mortality when accounting for differences in demographics, socioeconomic status, and comorbidities [6]. Notably, in the UK Black African men and women were over 2 times as likely to die from COVID-19 than those of White ethnicity [6]. Other large-scale epidemiological analyses from the UK demonstrated that those from South Asian, Black, and ‘Mixed’ ethnic groups had increased rates of COVID-19 death compared to the White group [2]. However, the evidence from other health systems such, as the United States, is conflicting [7], and data on COVID-19 cases and mortality in Canada by ethnicity are more limited [8, 9].

The mechanisms driving the inequalities are unclear but have been posited to be related to a complex and interrelated patterning of multiple factors, including medical factors – such as comorbidities and medication use – as well as social determinants, including cultural, behavioural, and occupational factors, and structural inequalities [10]. The presence of comorbidities has been associated with both a higher risk of SARS-CoV-2 infection and worse outcomes in individuals with COVID-19 [11, 12], while medications (i.e., certain glucose-lowering [13] or immune-modifying drugs [14]) have been linked to either an increased or reduced risk of COVID-19 outcomes. Among the social factors, poor living and working conditions, low income, health literacy, poverty, or exposure to air pollution have all been associated with COVID-19 infectivity and mortality [15–17]. Robustly ascertaining the comparative contributory effects has been difficult to untangle, and one study [18] has sought to quantify potential mediators, rather than reporting ‘overall’ effects [2, 4, 19–21].

Establishing a nuanced understanding of ethnic inequalities in COVID-19-related outcomes is needed to reduce the burden of COVID-19 and may permit rapid public health interventions should modifiable factors be identified. Here, we carried out two observational studies with harmonised designs to reduce the bias due to heterogeneous definitions of exposures, outcomes, and confounders, in the UK (England) and Ontario to quantify the associations between ethnicity and COVID-19 severity and explore potential modifiable and non-modifiable explanatory factors. We then sought to synthesise these cohort-level estimates using a meta-analysis.

## Methods

### Data sources and study population

#### QResearch

QResearch database (version 45) comprises individuals registered across 1321 general practices covering 18% of the English population with linkages of primary care data to hospitalisation, intensive care (ICU) admission, and mortality data. For this study, we included 9,828,099 adults aged 18 to 99 years contributing to the QResearch database with at least 12 months of continuous prior registration. The study period ran from the date of the first confirmed SARS-CoV-2 infection in England (24th January 2020, start of follow-up) until 31st October 2020 (end of follow-up), the occurrence of outcome, or death, whichever occurred earlier.

#### Ontario

The second data source is the population-level healthcare administrative data in Ontario, Canada’s most populous and most ethnically diverse province. These data include the entire population of Ontario (currently 14.5 million, representing nearly 40% of the Canadian population) and are linked to sociodemographic information, hospital, and ICU admissions; in this investigation, 10,273,496 people aged over 18 years were included. The study period ran from the 25th January 2020 (start of follow-up) to 30th September 2020 (end of follow-up), the occurrence of outcome, or death, whichever occurred earlier.

### Ethnicity and COVID-19 outcome: pooled analysis

In the first analysis, we explored the association between self-reported ethnicity and COVID-19 related death, hospitalisation, and ICU admission: these outcomes were slightly different in the QResearch and Ontario cohorts as based on country-specific definitions. For QResearch, outcomes included: (a) COVID-19 death, defined as either confirmed or suspected COVID-19 on death certificate, or a death from any cause with a confirmed positive SARS-CoV-2 test in the immediately preceding 28 days; (b) Hospitalisation due to COVID-19, defined as an admission with confirmed or suspected COVID-19 (as per ICD-10 codes U07.1 and U07.2), or new hospitalisation with a positive SARS-CoV-2 test in the immediately preceding 14 days; (c) ICU admission due to COVID-19, defined as admission to ICU with confirmed or suspected SARS-CoV-2 test in the preceding 28 days. In the Ontario database, outcomes were defined as: (a) COVID-19 death, defined as any death with a confirmed positive SARS-CoV-2 test in the immediately preceding 28 days; (b) Hospitalisation due to COVID-19, defined as an admission with confirmed or suspected COVID-19 (as per ICD-10 codes U07.1 and U07.2), or with a positive SARS-CoV-2 test between 28 days prior to and 14 days after the admission date; (c) ICU admission due to COVID-19, defined as a hospital admission that included ICU stay with confirmed or suspected COVID-19 (as per ICD-10 codes), or with a positive SARS-CoV-2 test between 28 days prior to and 14 days after the admission date.

We utilised a 3-level ethnicity classification comprised South Asian (Indian, Bangladeshi, Pakistani), Chinese, and ‘General Population’ (all other ethnic groups, 87.5% White in this cohort), based on the UK Office for National Statistics Census ethnic classification. In the Ontario linked healthcare administrative database, ethnicity was ascertained based on surnames, using lists that have been previously validated in this population to identify the two largest ethnic groups in Canada: South Asian and Chinese [22]. The positive predictive values for this approach to identifying ethnicity, when compared to self-reported ethnicity, are high: 89.3% for South Asians and 91.9% for Chinese; specificity 99.7% for both. People whose surnames were not on either list were labelled as ‘General population’ (all other ethnic groups, approximately 80% White). ‘General Population’ was used as the reference category for analyses.

The analyses were adjusted for demographic, clinical, and lifestyle factors (Supplementary Material; Tables S1 and S2); estimates of the associations between ethnicity and each of the three outcomes obtained in the QResearch and Ontario cohorts were combined in a two-stage random-effects meta-analysis. Further details on the definitions of the population and confounders are reported in the Supplementary Material.

### Percentage of excess risk mediated by risk factors

The contribution of possible ‘risk factor’ classes to the increased relative risks in different ethnic groups was quantified in the QResearch data as the ‘percentage of excess risk mediated’ (PERM) [23]. By evaluating the change in the magnitude of the exposure-outcome association in models with different confounders, this analysis helps clarify the extent to which a confounder (or a set of confounders) accounts for the association between ethnicity and COVID-19 outcome. For the PERM analyses, we defined 5-level ethnic groups as Mixed ethnicity, South Asian, Black, and ‘Other’ ethnic groups; hazard ratios (HRs), relative to White, were estimated separately for each of the three outcomes and the following set of confounders: ‘minimally adjusted’ model (age, sex, and region); household and social factors; comorbidities; lifestyle factors (including BMI); and ‘maximally adjusted’ model.

### Statistical analyses

Country-specific baseline socio-demographic and clinical characteristics were summarised using descriptive statistics by COVID-19 related hospitalisation, ICU admission, and mortality. In QResearch, survival analyses to evaluate the adjusted association of 3-level ethnicity with COVID-19 outcomes, accounting for clustering of practices (robust standard error), were performed with the Royston-Parmar model [24, 25]. We performed multiple imputation to replace missing values for ethnicity (20.2% missing), deprivation (0.6%), BMI (16.7%), and smoking status (5.2%) using chained equations under the missing at random assumption. These variables were modelled following a multinomial logistic model for ethnicity, ordinal logistic model for smoking and alcohol, and truncated regression for BMI. Results from five imputations were pooled using Rubin’s rules [26]. Complete case analyses and time-varying associations were performed as sensitivity analyses. Cox survival regressions were conducted to evaluate the adjusted associations of ethnicity with COVID-19 outcomes in the Ontario database; as the only missing data were a small number of people missing deprivation data (0.2%), we used complete cases regressions. The proportional hazards assumption was checked in both survival analyses by plotting log-log plot. HRs from maximally adjusted models were used as the common measure of association across QResearch and Ontario cohorts and combined with the DerSimonian-Laird random-effects method in a two-stage meta-analysis; heterogeneity was assessed with *I* ^2^.

In QResearch, we applied the following formula to estimate PERM using the HR across imputed datasets was:

$$\textrm{PERM}=100\frac{\ \left[\textrm{HR}\left(\textrm{age},\textrm{sex},\textrm{region}\right)\kern0.5em -\kern0.5em \textrm{HR}\left(\textrm{age},\textrm{sex},\textrm{region}+\textrm{risk}\ \textrm{factor}\ \textrm{group}\right)\right]}{\left[\textrm{HR}\left(\textrm{age},\textrm{sex},\textrm{region}\right)-1\right]\kern0.5em }$$

The PERM was also calculated for ‘maximal adjustment’ in each of the non-White ethnic groups to assess the extent to which inequalities were potentially attributable to the large set of measured adjustment factors.

All *p*-values are two sided and nominal statistical significance was considered at *p* < 0.05. We used Stata v.17 for the QResearch statistical analyses and SAS v.9.4 for the Ontario analyses. We followed current guidance for conducting and reporting observational studies using routinely collected health data (RECORD checklist in the **Supplementary Material**).

### Patient and public involvement reporting

Two public representatives advised on interest and appropriateness of the research questions, were involved in writing the protocol for the wider study, and input on lay-summaries describing the planned study.

## Results

### Study populations

In the QResearch cohort, there were 9,828,099 individuals; during follow-up, 11,597 COVID-19 deaths, 21,917 hospitalisations and 2932 ICU admissions occurred; in the Ontario cohort, corresponding figures were 10,273,496 individuals, 951 COVID-19 deaths, 5132 hospitalisations, and 1191 ICU admissions (Table 1). Ethnicity data and their classifications are summarised in Table 1 and characteristics stratified by ethnicity are provided in Tables S1-S2.

**Table 1:** Baseline sociodemographic, clinical characteristics and outcomes in the QResearch and Ontario cohorts

	QResearch				Ontario			
	Whole cohort	COVID-19 death	COVID-19 hospitalisation	COVID-19 ICU admission	Whole cohort	COVID-19 death	COVID-19 hospitalisation	COVID-19 ICU admission
Subjects (N)								
Age (yr)	9,828,099	11,597	21,917	2932	10,273,496	951	5132	1191
Mean (SD)	47.6 (18.8)	80.8 (11.7)	69.0 (18.0)	60.9 (13.8)	48.7 (18.5)	77.7 (12.9)	66.6 (18.0)	62.9 (14.1)
18-29	1,988,204 (20.2)	20 (0.2)	649 (3.0)	67 (2.3)	1,927,688 (18.8)	≤5 (≤0.5)	202 (3.9)	30 (2.5)
30-39	1,907,376 (19.4)	48 (0.4)	1067 (4.9)	160 (5.5)	1,764,197 (17.2)	6 (0.6)	260 (5.1)	44 (3.7)
40-49	1,620,268 (16.5)	125 (1.1)	1744 (8.0)	339 (11.6)	1,684,114 (16.4)	21 (2.2)	417 (8.1)	116 (9.7)
50-59	1,602,608 (16.3)	519 (4.5)	2881 (13.1)	711 (24.2)	1,834,451 (17.9)	53 (5.6)	815 (15.9)	263 (22.1)
60-69	1,196,452 (12.2)	1110 (9.6)	3402 (15.5)	822 (28.0)	1,509,133 (14.7)	152 (16.0)	992 (19.3)	336 (28.2)
70-79	922,198 (9.4)	2414 (20.8)	4401 (20.1)	611 (20.8)	973,829 (9.5)	206 (21.7)	980 (19.1)	262 (22.0)
80-89	471,167 (4.8)	4574 (39.4)	5485 (25.0)	204 (7.0)	464,253 (4.5)	332 (34.9)	1012 (19.7)	119 (10.0)
90-99	119,826 (1.2)	2787 (24.0)	2288 (10.4)	18 (0.6)	115,831 (1.1)	≥172 (≥18.1)	454 (8.8)	21 (1.8)
Sex								
Female	4,934,876 (50.2)	5154 (44.4)	9694 (44.2)	917 (31.3)	5,301,576 (51.6)	435 (45.7)	2429 (47.3)	440 (36.9)
Male	4,893,223 (49.8)	6443 (55.6)	12,223 (55.8)	2015 (68.7)	4,971,920 (48.4)	516 (54.3)	2703 (52.7)	751 (63.1)
Ethnicity (3 levels)								
General population ^a^	7,160,034 (72.9)	8955 (77.2)	16,597 (75.7)	2167 (73.9)	9,180,377 (89.4)	850 (89.4)	4649 (90.6)	1042 (87.5)
South Asian	585,810 (6.0)	582 (5.0)	1825 (8.3)	353 (12.0)	454,694 (4.4)	58 (6.1)	273 (5.3)	72 (6.0)
Chinese	96,391 (1.0)	32 (0.3)	77 (0.4)	15 (0.5)	638,425 (6.2)	43 (4.5)	210 (4.1)	77 (6.5)
Not recorded	1,985,864 (20.2)	2028 (17.5)	3418 (15.6)	397 (13.5)	–	–	–	–
Ethnicity (5 levels)								
White	6,264,009 (63.7)	8131 (70.1)	13,904 (63.4)	1603 (54.7)	–	–	–	–
Mixed	145,291 (1.5)	77 (0.7)	311 (1.4)	64 (2.2)	–	–	–	–
South Asian	585,810 (6.0)	582 (5.0)	1825 (8.3)	353 (12.0)	–	–	–	–
Black	407,604 (4.1)	550 (4.7)	1546 (7.1)	302 (10.3)	–	–	–	–
Other	439,521 (4.5)	229 (2.0)	913 (4.2)	213 (7.3)	–	–	–	–
Not recorded	1,985,864 (20.2)	2028 (17.5)	3418 (15.6)	397 (13.5)	–	–	–	–
Deprivation								
Quintile 1 – Least deprived	2,217,549 (22.6)	2462 (21.2)	4128 (18.8)	474 (16.2)	2,496,985 (24.3)	124 (13.0)	812 (15.8)	174 (14.6)
Quintile 2	2,143,852 (21.8)	2489 (21.5)	4371 (19.9)	521 (17.8)	2,152,782 (21.0)	154 (16.2)	832 (16.2)	191 (16.0)
Quintile 3	1,941,638 (19.8)	2650 (22.9)	4570 (20.9)	621 (21.2)	1,897,167 (18.5)	187 (19.7)	961 (18.7)	219 (18.4)
Quintile 4	1,792,050 (18.2)	2107 (18.2)	4460 (20.3)	599 (20.4)	1,813,916 (17.7)	198 (20.8)	958 (18.7)	263 (22.1)
Quintile 5 – Most deprived	1,676,662 (17.1)	1860 (16.0)	4311 (19.7)	711 (24.2)	1,912,646 (18.6)	288 (30.3)	1569 (30.6)	344 (28.9)
Not recorded	56,348 (0.6)	29 (0.3)	77 (0.4)	6 (0.2)	–	–	–	–
Home type								
Neither	9,749,068 (99.2)	8600 (74.2)	20,040 (91.4)	2884 (98.4)	–	–	–	–
Care home	60,971 (0.6)	2980 (25.7)	1819 (8.3)	37 (1.3)	67,212 (0.7)	334 (35.1)	862 (16.8)	92 (7.7)
Homeless	18,060 (0.2)	17 (0.1)	58 (0.3)	11 (0.4)	–	–	–	–
Household size								
1 person	3,490,475 (35.5)	4556 (39.3)	9472 (43.2)	1169 (39.9)	–	–	–	–
2 people	2,532,390 (25.8)	2361 (20.4)	5409 (24.7)	792 (27.0)	–	–	–	–
3-5 people	3,277,375 (33.3)	1401 (12.1)	4509 (20.6)	801 (27.3)	–	–	–	–
6-9 people	388,358 (4.0)	634 (5.5)	994 (4.5)	135 (4.6)	–	–	–	–
10 or more	139,501 (1.4)	2645 (22.8)	1533 (7.0)	35 (1.2)	–	–	–	–
Body mass Index (kg/m^2^)								
< 18.5	272,673 (2.8)	586 (5.1)	529 (2.4)	24 (0.8)	–	–	–	–
18.5-25	3,228,461 (32.8)	3728 (32.1)	5255 (24.0)	445 (15.2)	–	–	–	–
25-30	2,709,699 (27.6)	3401 (29.3)	6955 (31.7)	916 (31.2)	–	–	–	–
30-35	1,260,163 (12.8)	1739 (15.0)	4430 (20.2)	733 (25.0)	–	–	–	–
35-40	477,760 (4.9)	753 (6.5)	2012 (9.2)	397 (13.5)	–	–	–	–
≥ 40	239,768 (2.4)	394 (3.4)	1299 (5.9)	258 (8.8)	–	–	–	–
Not recorded	1,639,575 (16.7)	996 (8.6)	1437 (6.6)	159 (5.4)	–	–	–	–
Smoking								
Non-smoker	5,619,707 (57.2)	6077 (52.4)	12,252 (55.9)	1667 (56.9)	–	–	–	–
Ex-smoker	2,064,126 (21.0)	4499 (38.8)	7722 (35.2)	1014 (34.6)	–	–	–	–
Current smoker	1,633,590 (16.6)	742 (6.4)	1655 (7.6)	226 (7.7)	–	–	–	–
Not recorded	510,676 (5.2)	279 (2.4)	288 (1.3)	25 (0.9)	–	–	–	–
Comorbidities								
Asthma	1,342,685 (13.7)	1567 (13.5)	3749 (17.1)	482 (16.4)	1,607,620 (15.6)	178 (18.7)	1007 (19.6)	245 (20.6)
COPD	224,949 (2.3)	1578 (13.6)	2523 (11.5)	206 (7.0)	252,577 (2.5)	162 (17.0)	572 (11.1)	109 (9.2)
Hypertension	1,643,338 (16.7)	6941 (59.9)	10,876 (49.6)	1329 (45.3)	2,735,573 (26.6)	771 (81.1)	3269 (63.7)	713 (59.9)
Coronary heart disease	342,108 (3.5)	2640 (22.8)	3620 (16.5)	349 (11.9)	311,012 (3.0)	144 (15.1)	558 (10.9)	127 (10.7)
Stroke	208,496 (2.1)	2268 (19.6)	2736 (12.5)	137 (4.7)	81,829 (0.8)	70 (7.4)	277 (5.4)	42 (3.5)
Atrial fibrillation	234,637 (2.4)	2311 (19.9)	2863 (13.1)	162 (5.5)	179,709 (1.7)	162 (17.0)	567 (11.0)	87 (7.3)
Congestive cardiac failure	113,411 (1.2)	1519 (13.1)	1966 (9.0)	124 (4.2)	241,392 (2.3)	242 (25.4)	834 (16.3)	154 (12.9)
Diabetes	671,376 (6.8)	3593 (31.0)	6312 (28.8)	941 (32.1)	1,315,449 (12.8)	490 (51.5)	2062 (40.2)	510 (42.8)
Chronic kidney disease ^b^	383,449 (3.9)	3839 (33.1)	5026 (22.9)	398 (13.6)	267,379 (2.6)	246 (25.9)	878 (17.1)	218 (18.3)
Severe mental illness	1,091,954 (11.1)	2038 (17.6)	3850 (17.6)	455 (15.5)	–	–	–	–
Parkinson’s disease	25,054 (0.3)	421 (3.6)	450 (2.1)	14 (0.5)	–	–	–	–
Epilepsy	130,251 (1.3)	409 (3.5)	726 (3.3)	71 (2.4)	–	–	–	–
Dementia	98,591 (1.0)	3733 (32.2)	2614 (11.9)	26 (0.9)	171,844 (1.7)	392 (41.2)	1129 (22.0)	109 (9.2)
Rare neurological diseases	29,814 (0.3)	122 (1.1)	201 (0.9)	22 (0.8)	–	–	–	–
Learning disability	174,757 (1.8)	615 (5.3)	967 (4.4)	95 (3.2)	–	–	–	–
Cerebral palsy	10,892 (0.1)	26 (0.2)	62 (0.3)	10 (0.3)	–	–	–	–
Pulmonary hypertension/fibrosis	16,820 (0.2)	227 (2.0)	316 (1.4)	24 (0.8)	–	–	–	–
Rheumatoid arthritis/SLE ^c^	96,286 (1.0)	361 (3.1)	659 (3.0)	72 (2.5)	119,127 (1.2)	33 (3.5)	133 (2.6)	29 (2.4)
Liver cirrhosis/NAFLD	182,026 (1.9)	418 (3.6)	1097 (5.0)	185 (6.3)	53,399 (0.5)	24 (2.5)	115 (2.2)	29 (2.4)
Sickle cell disease	3546 (0.0)	7 (0.1)	36 (0.2)	9 (0.3)	2161 (0.0)	≤5 (≤0.5)	8 (0.2)	≤5 (≤0.4)
VTE/PVD	234,713 (2.4)	1669 (14.4)	2431 (11.1)	206 (7.0)	–	–	–	–
Cancer ^d^	69,259 (0.7)	545 (4.7)	765 (3.5)	96 (3.3)	3,102,868 (30.2)	493 (51.8)	2251 (43.9)	462 (38.8)
Immunosuppression	116,317 (1.2)	544 (4.7)	1079 (4.9)	191 (6.5)	–	–	–	–
Transplant (marrow/solid)	11,202 (0.1)	58 (0.5)	160 (0.7)	44 (1.5)	13,599 (0.1)	6 (0.6)	41 (0.8)	15 (1.3)
Crohn’s/colitis	–	–	–	–	84,287 (0.8)	≤5 (≤0.5)	35 (0.7)	9 (0.8)
HIV	–	–	–	–	19,272 (0.2)	≤5 (≤0.5)	21 (0.4)	≤5 (≤0.4)

^a^People not South Asian and Chinese - Ontario: approximately 80% White; QResearch; White, Other Asian, Black African, Black Caribbean, and Other

^b^Chronic kidney disease stage 3-5 in QResearch. ^c^ Rheumatoid arthritis alone in Ontario cohort. ^d^ Blood/respiratory cancer in QResearch, all cancer types in Ontario cohort

COPD – chronic obstructive pulmonary disease; SLE – systemic lupus erythematosus; NAFLD – Non-alcoholic fatty liver disease; VTE – venous thromboembolism; PVD – peripheral vascular disease; HIV – human immunodeficiency virus

Cells less than 5 are suppressed

### Cohort studies and meta-analyses

**Fig. 1:**
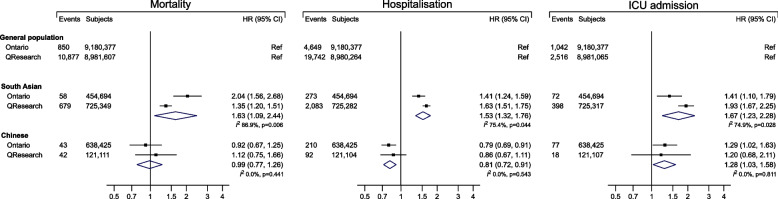
Cohort-level meta-analysis of individual participant data from QResearch and Ontario. Estimates and number of events and participants are shown following multiple imputation in QResearch cohort and for complete-case analysis in Ontario cohort. The reference ethnic group is “general population”, including: (1) people not South Asian and Chinese in Ontario (approximately 80% White); (2) White, Other Asian, Black African, Black Caribbean, and Other in QResearch

In QResearch, South Asian ethnicity was associated with increased rates of COVID-19 mortality (HR: 1.35; 95% CI: 1.20, 1.51; Fig. 1 and S1), hospitalisation (1.63; 1.51, 1.75; Fig. 1 and S2), and ICU admission (1.93; 1.67, 2.25; Fig. 1 and S3) compared to the general population group; corresponding estimates in Ontario were 2.04 (1.56, 2.68) for mortality, 1.41 (1.24, 1.59) for hospitalisation, and 1.41 (1.10, 1.79) for ICU admission. In the same maximally adjusted models, in QResearch there was no evidence of increased rates of COVID-19 mortality (HR: 1.12; 0.75, 1.66), hospitalisation (0.86; 0.67, 1.11), or ICU admission (1.20; 0.68, 2.11) in Chinese ethnic group compared to the general population group, whilst in Ontario the HRs were 0.92 (0.67, 1.25) for mortality, 0.79 (0.69, 0.91) for hospitalisation, and 1.29 (1.02, 1.63) for ICU admission. For all three outcomes, the direction of associations was similar for most of the confounders available in both the QResearch and Ontario cohorts, indicating an increased risk associated with the presence of medical conditions and a progressively higher risk in older people and larger households (Fig. S1-S3). In the QResearch cohort, complete case estimations were largely similar to those of the main analyses using multiple imputation (Fig. S4); time-varying associations by ethnic groups are presented in Fig. S5.

Combining estimates for South Asian ethnicity across QResearch and Ontario cohorts resulted in a random-effects HR of 1.63 (1.09, 2.44) for COVID-19 related mortality, with considerable heterogeneity between the two estimates (*I* ^2^ 86.9%; Fig. 1). Corresponding estimates for hospitalisation and ICU admission were 1.53 (1.32, 1.76) and 1.67 (1.23, 2.28), with considerable heterogeneity: *I* ^2^ 75.4% and *I*^2^ 74.9%, respectively. The pooled random-effects HRs comparing Chinese ethnicity to the general population were 0.99 (0.77, 1.26) for mortality, 0.81 (0.72, 0.91) for hospitalisation, and 1.28 (1.03, 1.58) for ICU admission; there was no evidence of heterogeneity for all three outcomes (*I*^2^ 0%; Fig. 1). There was no clear trend in the mortality, hospitalisation, or ICU admission HRs comparing ethnic groups across levels of deprivation (Fig. S6).

### Percentage of excess risk mediated by risk factor classes (QResearch)

The percentage of excess risk mediated by separate groups of potential attributable factors across the entirety of follow-up in QResearch is reported in Table 2. We estimated that approximately 20-30% of the excess risk of COVID-19-related hospitalisation in non-White ethnic groups may be mediated by household size/status and deprivation; and that differences in comorbidity prevalence may mediate up to approximately 20% of excess risk (in South Asian). For COVID-19-related ICU admission, adjustment for comorbidities accounted for up to approximately 30% of the excess risk, whereas maximal adjustment accounted for up to approximately 40% of the excess risk (in Black ethnic group). Differences in smoking habits and BMI did not appear to mediate any degree of excess risk of COVID-19-related death in any non-White ethnic group. Maximal adjustment accounted for 42.9% (South Asian) and 39.4% (Black) of the excess risks of death. Therefore, the majority of excess risk in non-White groups may not be accounted for the range of sociodemographic, lifestyle, and comorbidity factors considered in this analysis.

**Table 2:** Percentage of excess risk mediated in COVID-19-related outcomes in ethnic minority groups (QResearch cohort)

	Mixed race					
COVID-19-related death	HR	95% CI		PERM	95% CI	
Minimal adjustment	1.19	0.96	1.47	...	...	...
Household and social factors	1.08	0.87	1.33	57.9	-73.7	168.4
Comorbidities	1.11	0.90	1.37	42.1	-94.7	152.6
Lifestyle and BMI	1.22	0.98	1.50	...	...	...
Maximal adjustment	1.07	0.87	1.33	63.2	-73.7	168.4
**COVID-19-related hospitalisation**						
Minimal adjustment	1.83	1.63	2.06	...	...	...
Household and social factors	1.65	1.47	1.86	21.7	-3.6	43.4
Comorbidities	1.78	1.58	1.99	6.0	-19.3	30.1
Lifestyle and BMI	1.87	1.67	2.10	...	...	...
Maximal adjustment	1.68	1.49	1.88	18.1	-6.0	41.0
**COVID-19-related ICU admission**						
Minimal adjustment	2.58	2.00	3.32	...	...	...
Household and social factors	2.33	1.80	3.01	15.8	-27.2	49.4
Comorbidities	2.41	1.86	3.11	10.8	-33.5	45.6
Lifestyle and BMI	2.68	2.07	3.45	...	...	...
Maximal adjustment	2.35	1.82	3.04	14.6	-29.1	48.1
**South Asian**						
**COVID-19-related death**	**HR**	**95% CI**		**PERM**	**95% CI**	
Minimal adjustment	1.56	1.40	1.74	...	...	...
Household and social factors	1.32	1.18	1.48	42.9	14.3	67.9
Comorbidities	1.41	1.27	1.58	26.8	-3.6	51.8
Lifestyle and BMI	1.64	1.47	1.83	...	...	...
Maximal adjustment	1.32	1.17	1.48	42.9	14.3	69.6
**COVID-19-related hospitalisation**						
Minimal adjustment	2.08	1.96	2.22	...	...	...
Household and social factors	1.82	1.71	1.93	24.1	13.9	34.3
Comorbidities	1.87	1.75	1.99	19.4	8.3	30.6
Lifestyle and BMI	2.22	2.08	2.36	...	...	...
Maximal adjustment	1.78	1.66	1.90	27.8	16.7	38.9
**COVID-19-related ICU admission**						
Minimal adjustment	2.82	2.47	3.23	...	...	...
Household and social factors	2.52	2.21	2.87	16.5	-2.7	33.5
Comorbidities	2.25	1.95	2.58	31.3	13.2	47.8
Lifestyle and BMI	3.14	2.75	3.60	...	...	...
Maximal adjustment	2.38	2.07	2.73	24.2	4.9	41.2
**Black**						
**COVID-19-related death**	**HR**	**95% CI**		**PERM**	**95% CI**	
Minimal adjustment	1.71	1.54	1.90	...	...	...
Household and social factors	1.41	1.27	1.56	42.3	21.1	62.0
Comorbidities	1.55	1.39	1.72	22.5	-1.4	45.1
Lifestyle and BMI	1.78	1.60	1.98	...	...	...
Maximal adjustment	1.43	1.28	1.59	39.4	16.9	60.6
**COVID-19-related hospitalisation**						
Minimal adjustment	2.20	2.06	2.34	...	...	...
Household and social factors	1.83	1.72	1.94	30.8	21.7	40.0
Comorbidities	2.06	1.93	2.19	11.7	0.8	22.5
Lifestyle and BMI	2.10	1.97	2.24	8.3	-3.3	19.2
Maximal adjustment	1.75	1.65	1.86	37.5	28.3	45.8
**COVID-19-related ICU admission**						
Minimal adjustment	2.95	2.59	3.35	...	...	...
Household and social factors	2.49	2.19	2.84	23.6	5.6	39.0
Comorbidities	2.57	2.25	2.94	19.5	0.5	35.9
Lifestyle and BMI	2.71	2.38	3.10	12.3	-7.7	29.2
Maximal adjustment	2.19	1.91	2.52	39.0	22.1	53.3
**Other ethnic group**						
**COVID-19-related death**	**HR**	**95% CI**		**PERM**	**95% CI**	
Minimal adjustment	1.13	0.98	1.31	...	...	...
Household and social factors	1.03	0.89	1.19	76.9	-46.2	184.6
Comorbidities	1.20	1.03	1.39	...	...	...
Lifestyle and BMI	1.18	1.01	1.37	...	...	...
Maximal adjustment	1.14	0.99	1.33	...	...	...
**COVID-19-related hospitalisation**						
Minimal adjustment	1.68	1.56	1.81	...	...	...
Household and social factors	1.51	1.40	1.63	25.0	7.4	41.2
Comorbidities	1.75	1.62	1.89	...	...	...
Lifestyle and BMI	1.85	1.71	1.99	...	...	...
Maximal adjustment	1.71	1.59	1.85	...	...	...
**COVID-19-related ICU admission**						
Minimal adjustment	2.54	2.16	2.98	...	...	...
Household and social factors	2.30	1.96	2.70	15.6	-10.4	37.7
Comorbidities	2.43	2.07	2.86	7.1	-20.8	30.5
Lifestyle and BMI	3.01	2.56	3.53	...	...	...
Maximal adjustment	2.61	2.22	3.07	...	...	...

Percentage of excess risk mediated (PERM; relative to White adults) by distinct classes of confounders by ethnic groups during the study period. These are compared to a minimally adjusted model, which accounted for age, sex and geographical region. Results denote hazard ratios with 95% confidence intervals derived from flexible parametric survival models in the multiple imputed database (9,828,099 individuals; 11597 deaths; 21917 hospitalisations; 2932 ICU admissions).

## Discussion

In this international study of population-level healthcare databases covering over 20 million individuals, we showed that adults of South Asian background had a 63% increased risk of COVID-19 mortality, 53% increased risk of COVID-19-related hospital admission, and 67% increased risk of ICU admission overall compared to the general population in England and Ontario. This compares to 28% of increased risk of ICU admission in Chinese, with no evidence of increased mortality and hospitalisation risks. In England, sociodemographic, lifestyle, and clinical factors accounted for approximately 40% of excess risks of COVID-19 death.

Our results are consistent with other UK population-level analyses derived from data using combinations of different IT systems, which also reported similar estimates of risk in non-White ethnic groups [2]. In this respect, it is important to note that the risks of COVID-19 outcomes estimated in QResearch across ethnic groups, and combined with the results from Ontario, should be considered in view of some variations in the magnitude of associations between ethnicity and COVID-19 outcomes both between waves and within the same wave; more importantly, the public health implications of these variations are primarily determined by the country- and region-specific change in the absolute risk of each outcome over time [27].

Whilst it is increasingly established in the literature that non-white ethnicity is associated with increased risk of severe COVID-19 outcomes, the degree to which modifiable and other factors may contribute to this risk in different ethnic groups is poorly understood. Some ethnic communities may be disadvantaged as living in poorer socioeconomic environments where the risk of infection and worse outcomes is higher, including overcrowded multigenerational houses or occupations with a high degree of public contact [2, 18]; at the same time, biological factors have been suggested to play a role as well, such as an unfavourable metabolic-inflammatory milieu (i.e., obesity, multimorbidity) [11, 20, 28]. In our investigation, rather than reporting summary effect estimates after full or serial adjustment, our approach in the QResearch also included assessment of relative contribution of potential attributable factors and suggests that there may be heterogeneity in the mechanistic underpinnings the increased risks in different ethnic groups. Our study found that the sociodemographic, lifestyle, and clinical factors considered in this investigation accounted for approximately 40% of excess risks of COVID-19 death. Hence, further research should investigate whether other factors, not captured in our data, may explain the proportion of excess risks in some ethnic groups and possible causal pathways in the COVID-19 syndemic [29]. It is possible, in fact, that ethnic differences are at least in part the epiphenomenon of a complex network of other risk factors associated with a higher risk of COVID-19 outcomes, including overcrowding and occupation [30].

Our study analysed in greater detail the differential effects of deprivation within ethnic groups, as well as the relative contributions of different factors to the increased risks in non-White groups, given the suggested interplay between ethnicity and deprivation on the risk of COVID-19 outcomes [31]. Our results also expand and clarify the evidence base regarding ethnic inequalities in COVID-19 outcomes in several ways. First, in contrast to evidence generated using data only from those attending hospitals or registered with providers within fragmented healthcare systems investigating the role of sociodemographic and clinical characteristics on the risk of outcomes across ethnic groups [27, 32], our population-level approach examined the relevant risk trajectories and avoided conditioning on positive tests or other intermediates [33]. Second, much of the available evidence about ethnicity and COVID-19 related outcomes is highly heterogeneous in terms of study designs, population, definitions of outcomes/exposures, confounders adjusted for (if any), and settings (geographical and healthcare system). This negatively affected individual study interpretation but also limited the cohesive synthesis of evidence via meta-analytical approaches due to significant within- and between-study heterogeneity [4, 34]. We explicitly sought to harmonise analytical approaches to facilitate pooling of robust estimates from multiple geographical units, namely different nations (England and Canada). Other key strengths of our study include the use of two large, population-level and representative healthcare databases without selection bias, which possess individual-level linkages across the healthcare network enabling accurate ascertainment of exposures, confounders, and outcomes. Our flexible harmonisation of definitions and analytical approaches facilitated cohort-level meta-analysis of results from both main study databases; we also used the Royston-Parmar survival model which allowed us to explore whether the association between ethnicity and COVID-19 related outcomes changed across the first and second wave. Lastly, we investigated the possible mediation role of some factors in explaining the increased risk observed across ethnic groups in UK. In this respect, it should be noted that different methods exist to investigate mediation (including the possibility to account for intermediate confounding) [35]; furthermore, while the difference between a confounder and mediator is well-known, the same factors may be considered mediators in some context and confounders in others, or even in the same context by different investigators [36], further highlighting the complex interactions among multiple factors in determining the health status. Moreover, some potential mediators have not been included in our analyses (i.e., education, employment status, income). As such, our PERM results should be considered explorative and no definitive causal inference can be derived from them: it is plausible that the comparative causal role of these factors would be different in heterogeneous healthcare systems and societies. Our study has also some limitations, including the inability to further disaggregate ethnicity into more granular groups in Ontario; lack of recorded other information that may be relevant to disease risks (such as occupation, which is relevant to SARS-CoV-2 exposure, and detailed household composition) [37]; the risk of residual confounding, which affects every observational analysis and hampers a conclusive causal interpretation; missing data, which were addressed assuming a missing at random mechanism, yet previous evidence would indicate that ethnicity could be missing not at random: [38] however, the complete-case analysis for the latter scenario [39] resulted in estimates virtually identical to those obtained using multiple imputation; and the potential variations in the ascertainment of COVID-19 infections over time, between countries, and among ethnic groups [40]. Furthermore, the contribution of potential attributable factors was explored only in the QResearch cohort as several of these factors were not available in the Ontario administrative data.

Evidence from large-scale cohort studies in England and Canada and from meta-analyses provide robust evidence of ethnic inequalities in COVID-19 outcomes. Not only do these persist despite accounting for potential sociodemographic and clinical confounders but the risks in individual ethnic groups have varied during the pandemic. The currently unexplainable proportion of excess risks in non-White groups requires careful consideration of economic, healthcare system, and other factors to guide public health strategy to protect everyone as the pandemic progresses globally.

## Supplementary Information

**Additional file 1.**

## Data Availability

Public access to the databases is closed. To guarantee the confidentiality of personal and health information, only the authors have had access to the data during the study in accordance with the relevant license agreements. Ontario: The dataset from this study is held securely in coded form at ICES. While legal data sharing agreements between ICES and data providers (e.g., healthcare organizations and government) prohibit ICES from making the dataset publicly available, access may be granted to those who meet pre-specified criteria for confidential access, available at https://www.ices.on.ca/DAS. QResearch: Access to QResearch data is according to the information on the QResearch website (www.qresearch.org).
