# Supplementary material for: Ethnic disparities in COVID-19 outcomes: a multinational cohort study of 20 million individuals from England and Canada
Source: BMC Public Health. 2023 Feb 27;23:399. doi: 10.1186/s12889-023-15223-8 (PMC9969387; doi:10.1186/s12889-023-15223-8)
Supplement: Supplementary file 1 — Additional file 1. [file 12889_2023_15223_MOESM1_ESM.pdf]

SUPPLEMENTARY MATERIAL

Ethnic disparities in COVID-19 outcomes: a multinational cohort study of 20 million individuals from England and Canada

SUPPLEMENTARY METHODS

Data sources and study population

QResearch

The QResearch database (version 45) comprises individuals registered across 1,321 general practices using the Egton Medical Information System (EMIS) software in England, covering 18% of the English population. Anonymised primary care medical records from consenting practices are linked to hospital admission data (including intensive care), mortality data (through the Office for National Statistics), and SARS-CoV-2 national infectious disease surveillance system provided by Public Health England. For this study, we included 9,828,099 adults aged 18 to 99 years contributing to the QResearch database with at least 12 months of continuous prior registration. The study period was the date of the first confirmed SARS-CoV-2 infection in the UK (24th January 2020) to 31st October 2020.

Ontario

The population-level healthcare administrative data in Ontario combines the following datasets: Registered Persons Database (RPDB), which includes sociodemographic information; Discharge Abstracts Database (DAD) on all admissions to acute care hospitals as well as ITU attendances; National Ambulatory Care Reporting Service (NACRS), which includes detailed information on all hospital emergency department visits; Ontario Health Insurance Plan (OHIP) for all fee-for-service claims for inpatient and ambulatory care, diagnostic and therapeutic procedures; and the Ontario Laboratory Information System (OLIS), with information on biochemistry, haematology, microbiology and SARS-CoV-2 laboratory test results from all private, hospital and public health laboratories. These datasets were linked using unique encoded identifiers and analysed at ICES (formerly the Institute for Clinical Evaluative Sciences), an independent non-profit research institute. For this study, we included 10,273,496 people aged over 18 years registered to the database on the 25th January 2020 who had at least 1 year of prior healthcare eligibility. The study excluded people living in rural areas, as relatively few ethnic minority people live in these areas. The study period was 25th January 2020 to 30th September 2020.

Potential confounders

The available confounders included in the statistical models differed between the QResearch (**Table S1**) and Ontario (**Table S2**) database. The selection of confounders was based on theoretical considerations[1] and empirical evidence from studies investigating the association between ethnicity and COVID-19 outcomes.[2-5]

QResearch

For the QResearch database, we included the sociodemographic variables: age, sex, deprivation (defined by fifths of the Townsend deprivation, a census-based measure of material deprivation based on a standardised score combining area-

level information on count of households without a car, overcrowded households, households not owner-occupied, and persons unemployed),[6] type of residence (care home, homeless, or neither), and household size (count of patients at a particular household ID among those registered at the practice). Lifestyle variables included body mass index (BMI) and smoking status (current smoker, ex-smoker or non-smoker). The following co-morbidities were included at study entry: asthma, chronic obstructive pulmonary disease (COPD), hypertension, coronary heart disease, stroke, atrial fibrillation (AF), congestive cardiac failure (CCF), diabetes, chronic kidney disease (stages 3-5), severe mental illness, Parkinson's disease, epilepsy, dementia, rare neurological diseases, learning disability, cerebral palsy, pulmonary hypertension/fibrosis, rheumatoid arthritis/systemic lupus erythematosus (SLE), liver diseases (cirrhosis/non-alcoholic fatty liver disease (NALFD), sickle cell disease, venous thromboembolism (VTE)/peripheral vascular disease (PVD), cancer (blood/respiratory), immunosuppression, solid/bone marrow transplant. The clinical codes in the primary care and hospital records used to identify the medical conditions and medications are available at: https://www.qresearch.org/data/qcode-group-library/.

Ontario

In the Ontario linked healthcare administrative database, sociodemographic variables included: age, sex, deprivation (defined by the material deprivation quintile measure from the Ontario Marginalization Index; the material deprivation indicator is based on the proportions of: (a) the population aged 20+ without a high-school diploma; (b) families who are lone parent families; (c) total income from government transfer payments for population aged 15+; (d) the population aged 15+ who are unemployed; (e) the population considered low-income; (f) households living in dwellings that are in need of major repair),[7] and residence in a long-term care facility such as nursing home/care home. Co-morbidities included: asthma, COPD, hypertension, coronary heart disease, cerebrovascular disease, atrial fibrillation, congestive heart failure, diabetes, chronic kidney disease, dementia, rheumatoid arthritis, liver disease, sickle cell disease, cancer, solid organ or bone marrow transplant, inflammatory bowel disease, and HIV. Lifestyle data including BMI and smoking status are not available in Ontario administrative data. The clinical codes used to identify the medical conditions are those reported in the table below or in the related reference:

Asthma	Gershon AS et al. Can Respir J 2009;16:183-8
COPD	Gershon AS et al. COPD 2009;6(5):388-94
Hypertension	Tu K et al. Open Med 2007;1(1):18-26.
Coronary heart disease	ICD-10 codes I20, I21, I22, I25 in prior 5 years; *OR* CCP codes 48.02, 48.03, 48.1 *OR* CCI codes 1.IJ.50, 1.IJ.57, 1.IJ.76 in prior 20 years
Stroke	ICD-10 codes G45.0, G45.1, G45.2, G45.3, G45.8, G45.9, H34.0, H34.1, I63, I64 in prior 5 years
Atrial fibrillation	ICD-9 code 427.3 *OR* ICD-10 code I48 in prior 20 years
CHF	Schultz SE et al. Chronic Dis Inj Can 2013;33(3):160-6
Diabetes	Lipscombe LL et al. BMC Health Serv Res 2018;18:316
CKD	Fleet JL et al. BMC Nephrol 2013;14:81
Dementia	Jaakkimainen RL et al. J Alzheim Dis 2016;54:337-49
Rheumatoid arthritis	Widdifield J et al. BMC Musculoskel Dis 2014;15(1):216
Liver cirrhosis	Lapointe-Shaw L et al. PLoS One 2018;13(8):e0201120
Sickle cell disease	ICD-9 code 282.6 *OR* ICD-10 code D57.0, D57.1, D57.2, D57.8 in prior 20 years
Cancer	ICD-9 code 140–239 *OR* ICD-10 code C01–C99 in prior 20 years
Transplant	Record in solid organ transplant registry prior to Dec 2018; *OR* CCI codes 1.PC.85, 1.HZ.85, 1.GR.85, 1.GT.85, 1.HY.85, 1.OA.85, 1.OK.85, 1.OB.85 after Jan 2019; *OR* CCP code 53.0 or CCI code 1.WY.19, 1.LZ.19 in prior 20 years
Crohn's and colitis	Benchimol EI et al. J Clin Epi 2014;67(8):887-96
HIV	Gershon AS et al. PLoS One 2011;6(6):e21748

ICD-9 = International Classification of Diseases, 9th edition; ICD-10 = International Classification of Diseases, 10th edition
CCP = Canadian Classifications of Diagnostic, Therapeutic and Surgical Procedures; CCI = Canadian Classification of Health Interventions

REFERENCES

1. VanderWeele TJ. Principles of confounder selection. *European Journal of Epidemiology* 2019;34(3):211-19. doi: 10.1007/s10654-019-00494-6

2. Sze S, Pan D, Nevill CR, et al. Ethnicity and clinical outcomes in COVID-19: A systematic review and meta-analysis. *EClinicalMedicine* 2020;29:100630. doi: 10.1016/j.eclinm.2020.100630 [published Online First: 2020/11/18]

3. Mathur R, Rentsch CT, Morton CE, et al. Ethnic differences in SARS-CoV-2 infection and COVID-19-related hospitalisation, intensive care unit admission, and death in 17 million adults in England: an observational cohort study using the OpenSAFELY platform. *Lancet* 2021;397(10286):1711-24. doi: 10.1016/S0140-6736(21)00634-6 [published Online First: 2021/05/04]

4. Rentsch CT, Kidwai-Khan F, Tate JP, et al. Patterns of COVID-19 testing and mortality by race and ethnicity among United States veterans: A nationwide cohort study. *PLOS Medicine* 2020;17(9):e1003379. doi: 10.1371/journal.pmed.1003379

5. Yates T, Summerfield A, Razieh C, et al. A population-based cohort study of obesity, ethnicity and COVID-19 mortality in 12.6 million adults in England. *Nat Commun* 2022;13(1):624. doi: 10.1038/s41467-022-28248-1 [published Online First: 2022/02/04]

6. Townsend P, Phillimore P, Beattie A. Health and deprivation: inequality and the North Croom Helm: London. *Health Policy* 1988;10:207-06.

7. 2016 Ontario Marginalization Index: Public Health Ontario; [Available from: https://www.publichealthontario.ca/-/media/documents/O/2017/on-marg-userguide.pdf accessed 22 May 2021].

Table S1: Baseline characteristics by ethnic groups (QResearch cohort)

Characteristics		General population	South Asian	Chinese
**Subjects (N)**		7160034	585810	96391
**Age (yr)**	Mean (SD)	48.8 (18.6)	42.2 (15.5)	36.8 (14.9)
	18-29	1276233 (17.8)	123062 (21.0)	39991 (41.5)
	30-39	1368700 (19.1)	169519 (28.9)	24230 (25.1)
	40-49	1194140 (16.7)	134322 (22.9)	13938 (14.5)
	50-59	1214588 (17.0)	70854 (12.1)	8339 (8.7)
	60-69	919757 (12.8)	48972 (8.4)	5974 (6.2)
	70-79	734443 (10.3)	24093 (4.1)	2667 (2.8)
	80-89	361985 (5.1)	13050 (2.2)	1073 (1.1)
	90-99	90188 (1.3)	1938 (0.3)	179 (0.2)
**Sex**	Female	3713017 (51.9)	277696 (47.4)	52109 (54.1)
	Male	3447017 (48.1)	308114 (52.6)	44282 (45.9)
**Deprivation**	Quintile 1 (least deprived)	1637140 (22.9)	48894 (8.3)	8851 (9.2)
	Quintile 2	1566234 (21.9)	73845 (12.6)	11794 (12.2)
	Quintile 3	1399191 (19.5)	121025 (20.7)	13612 (14.1)
	Quintile 4	1286957 (18.0)	164561 (28.1)	20284 (21.0)
	Quintile 5 (most deprived)	1229106 (17.2)	174538 (29.8)	40156 (41.7)
	Not recorded	41406 (0.6)	2947 (0.5)	1694 (1.8)
**Household size**	1 person	2623540 (36.6)	149168 (25.5)	42617 (44.2)
	2 people	1933609 (27.0)	96118 (16.4)	16816 (17.4)
	3-5 people	2278888 (31.8)	251363 (42.9)	25820 (26.8)
	6-9 people	230175 (3.2)	77453 (13.2)	4823 (5.0)
	10 or more	93822 (1.3)	11708 (2.0)	6315 (6.6)
**Home type**	Neither	7094991 (99.1)	583673 (99.6)	96260 (99.9)
	Care home	49493 (0.7)	1418 (0.2)	112 (0.1)
	Homeless	15550 (0.2)	719 (0.1)	19 (0.0)
**Body mass index (kg/m^2)**	<18.5	185678 (2.6)	22618 (3.9)	7335 (7.6)
	18.5-25	2458957 (34.3)	201759 (34.4)	50574 (52.5)
	25-30	2110485 (29.5)	183205 (31.3)	14168 (14.7)
	30-35	998630 (13.9)	72090 (12.3)	2656 (2.8)
	35-40	382674 (5.3)	21746 (3.7)	555 (0.6)
	≥40	194536 (2.7)	8299 (1.4)	222 (0.2)
	Not recorded	829074 (11.6)	76093 (13.0)	20881 (21.7)
**Smoking**	Non-smoker	4060877 (56.7)	445549 (76.1)	76445 (79.3)
	Ex-smoker	1661661 (23.2)	49342 (8.4)	7372 (7.6)
	Current smoker	1240714 (17.3)	67022 (11.4)	7774 (8.1)
	Not recorded	196782 (2.7)	23897 (4.1)	4800 (5.0)
**Comorbidities**	Asthma	1040959 (14.5)	68826 (11.7)	4164 (4.3)
	COPD	190894 (2.7)	4301 (0.7)	193 (0.2)
	Hypertension	1294592 (18.1)	82177 (14.0)	5253 (5.4)
	Coronary heart disease	265163 (3.7)	20837 (3.6)	616 (0.6)
	Stroke	163596 (2.3)	7284 (1.2)	380 (0.4)
	Atrial fibrillation	187481 (2.6)	3349 (0.6)	365 (0.4)
	Congestive cardiac failure	90070 (1.3)	4527 (0.8)	151 (0.2)
	Diabetes	501791 (7.0)	77142 (13.2)	2925 (3.0)
	Chronic kidney disease	303188 (4.2)	15990 (2.7)	715 (0.7)
	Severe mental illness	870409 (12.2)	33269 (5.7)	2202 (2.3)
	Parkinson's disease	19713 (0.3)	776 (0.1)	65 (0.1)
	Epilepsy	103177 (1.4)	4802 (0.8)	182 (0.2)
	Dementia	78066 (1.1)	2977 (0.5)	154 (0.2)
	Rare neurological diseases	23650 (0.3)	644 (0.1)	42 (0.0)
	Learning disability	134687 (1.9)	7781 (1.3)	355 (0.4)
	Cerebral palsy	8161 (0.1)	495 (0.1)	16 (0.0)
	Pulmonary hypertension/fibrosis	13444 (0.2)	766 (0.1)	41 (0.0)
	Rheumatoid arthritis/SLE	75355 (1.1)	5510 (0.9)	352 (0.4)
	Liver cirrhosis/NAFLD	139525 (1.9)	18824 (3.2)	1046 (1.1)
	Sickle cell disease	3059 (0.0)	110 (0.0)	<5
	VTE/PVD	188339 (2.6)	6344 (1.1)	205 (0.2)
	Cancer (blood & respiratory)	54520 (0.8)	2118 (0.4)	220 (0.2)
	Immunosuppression	89448 (1.2)	5406 (0.9)	418 (0.4)
	Transplant	8270 (0.1)	780 (0.1)	52 (0.1)

COPD – chronic obstructive pulmonary disease; SLE – systemic lupus erythematosus; NAFLD – Non-alcoholic fatty liver disease; VTE – venous thromboembolism; PVD – peripheral vascular disease. Cells less than 5 are suppressed.

Table S2: Baseline characteristics by ethnic groups (Ontario cohort)

Characteristics		General population	South Asian	Chinese
**Subjects (N)**		9180377	454694	638425
**Age (years)**	Mean (SD)	48.9 (18.6)	45.6 (17.3)	48.3 (17.8)
	18-29	1722979 (18.8%)	90393 (19.9%)	114316 (17.9%)
	30-39	1546755 (16.8%)	102788 (22.6%)	114654 (18.0%)
	40-49	1487156 (16.2%)	87984 (19.4%)	108974 (17.1%)
	50-59	1639115 (17.9%)	68922 (15.2%)	126414 (19.8%)
	60-69	1365884 (14.9%)	52798 (11.6%)	90451 (14.2%)
	70-79	886902 (9.7%)	35728 (7.9%)	51199 (8.0%)
	80-89	423745 (4.6%)	13859 (3.0%)	26649 (4.2%)
	90-99	107841 (1.2%)	2222 (0.5%)	5768 (0.9%)
**Sex**	Female	4735518 (51.6%)	225753 (49.6%)	340305 (53.3%)
	Male	4444859 (48.4%)	228941 (50.4%)	298120 (46.7%)
**Deprivation**	Quintile 1 (least deprived)	2241122 (24.4%)	84292 (18.5%)	171571 (26.9%)
	Quintile 2	1901861 (20.7%)	87244 (19.2%)	163677 (25.6%)
	Quintile 3	1678636 (18.3%)	109029 (24.0%)	109502 (17.2%)
	Quintile 4	1613406 (17.6%)	101217 (22.3%)	99293 (15.6%)
	Quintile 5 (most deprived)	1745352 (19.0%)	72912 (16.0%)	94382 (14.8%)
**Home type**	Long-term care resident	63810 (0.7%)	767 (0.2%)	2635 (0.4%)
**Comorbidities**	Asthma	1487766 (16.2%)	64143 (14.1%)	55711 (8.7%)
	COPD	242774 (2.6%)	3848 (0.8%)	5955 (0.9%)
	Hypertension	2484081 (27.1%)	116975 (25.7%)	134517 (21.1%)
	Coronary heart disease	290147 (3.2%)	14535 (3.2%)	6330 (1.0%)
	Stroke	76964 (0.8%)	2347 (0.5%)	2518 (0.4%)
	Atrial fibrillation	171981 (1.9%)	3256 (0.7%)	4472 (0.7%)
	Congestive cardiac failure	227978 (2.5%)	7626 (1.7%)	5788 (0.9%)
	Diabetes	1157417 (12.6%)	87099 (19.2%)	70933 (11.1%)
	Chronic kidney disease	243520 (2.7%)	12004 (2.6%)	11855 (1.9%)
	Dementia	161180 (1.8%)	3998 (0.9%)	6666 (1.0%)
	Rheumatoid arthritis	110617 (1.2%)	5144 (1.1%)	3366 (0.5%)
	Liver cirrhosis	49932 (0.5%)	1559 (0.3%)	1908 (0.3%)
	Sickle cell disease	2132 (0.0%)	18 (0.0%)	11 (0.0%)
	Cancer (solid organ/hematopoietic)	2858051 (31.1%)	89299 (19.6%)	155518 (24.4%)
	Transplant (marrow/solid)	12505 (0.1%)	510 (0.1%)	584 (0.1%)
	Crohn’s & colitis	80328 (0.9%)	2850 (0.6%)	1109 (0.2%)
	HIV	18440 (0.2%)	350 (0.1%)	482 (0.1%)

COPD – chronic obstructive pulmonary disease; HIV – human immunodeficiency virus.

Figure S1: Adjusted hazard ratios for COVID-19 death in QResearch and Ontario cohorts

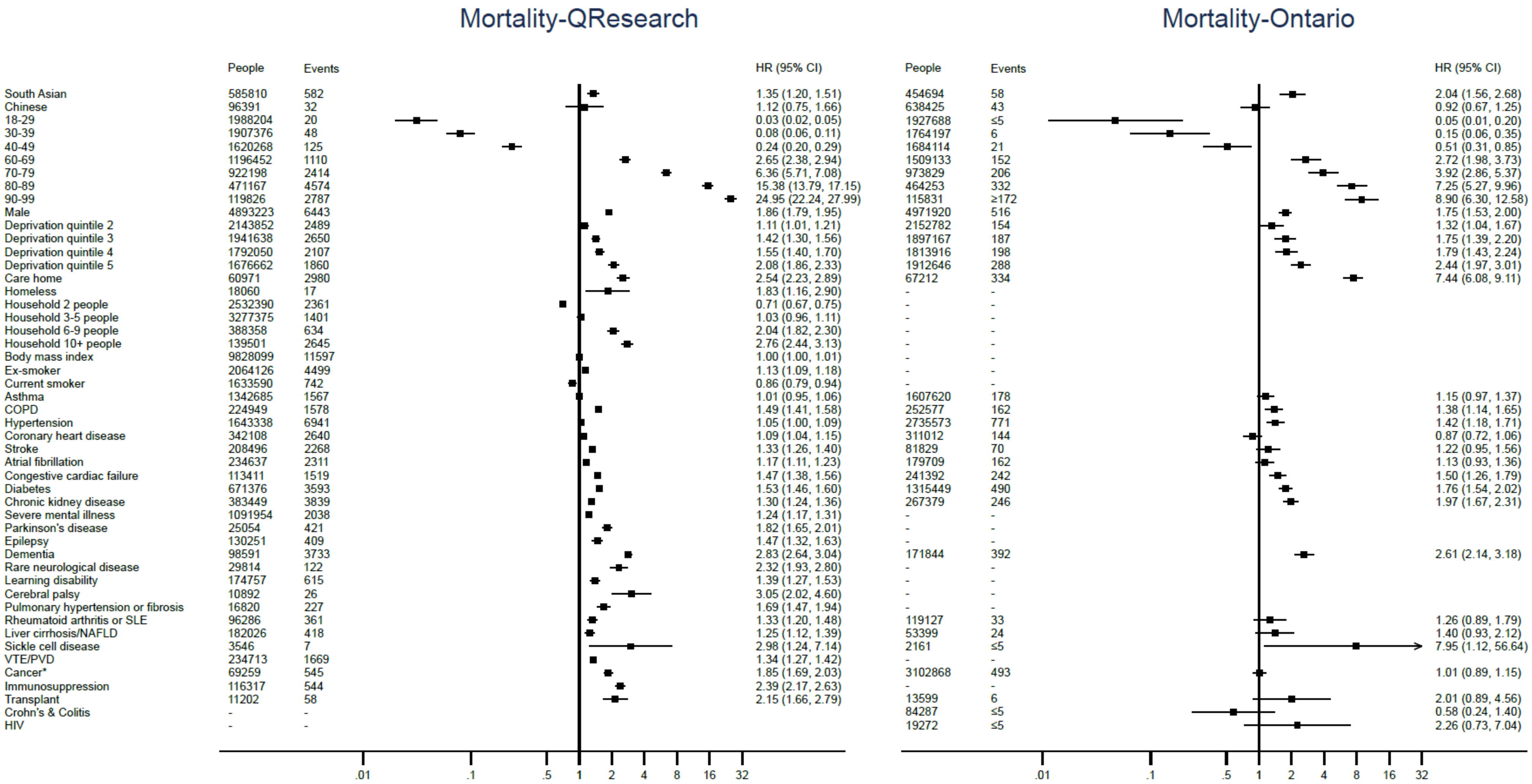

Number of events and participants refer to complete-case data (Table 1, Table S1 and Table S2)

Rheumatoid arthritis alone in Ontario cohort. *Blood/respiratory cancer in QResearch, all cancer types in Ontario cohort.
The reference ethnic group is "general population": people not South Asian and Chinese - Ontario: approximately 80% White; QResearch; White, Other Asian, Black African, Black Caribbean, and Other. COPD – chronic obstructive pulmonary disease; SLE – systemic lupus erythematosus; NAFLD – Non-alcoholic fatty liver disease; VTE – venous thromboembolism; PVD – peripheral vascular disease; HIV – human immunodeficiency virus. Reference groups are: Ethnicity – White; Age – 50-59 years; Sex – Female; Deprivation – 1st quintile (least deprived); Residence – neither (QResearch), no long-term care resident (Ontario); Household size – 1 person; Smoking status – non-smoker. For body mass index (BMI, kg/m^2), hazard ratio is reported per 1-unit increase. For all comorbidities, reference is no disease (for chronic kidney disease [CKD] it is CKD stage 1-2)

Figure S2: Adjusted hazard ratios for COVID-19 hospitalisation in QResearch and Ontario cohorts

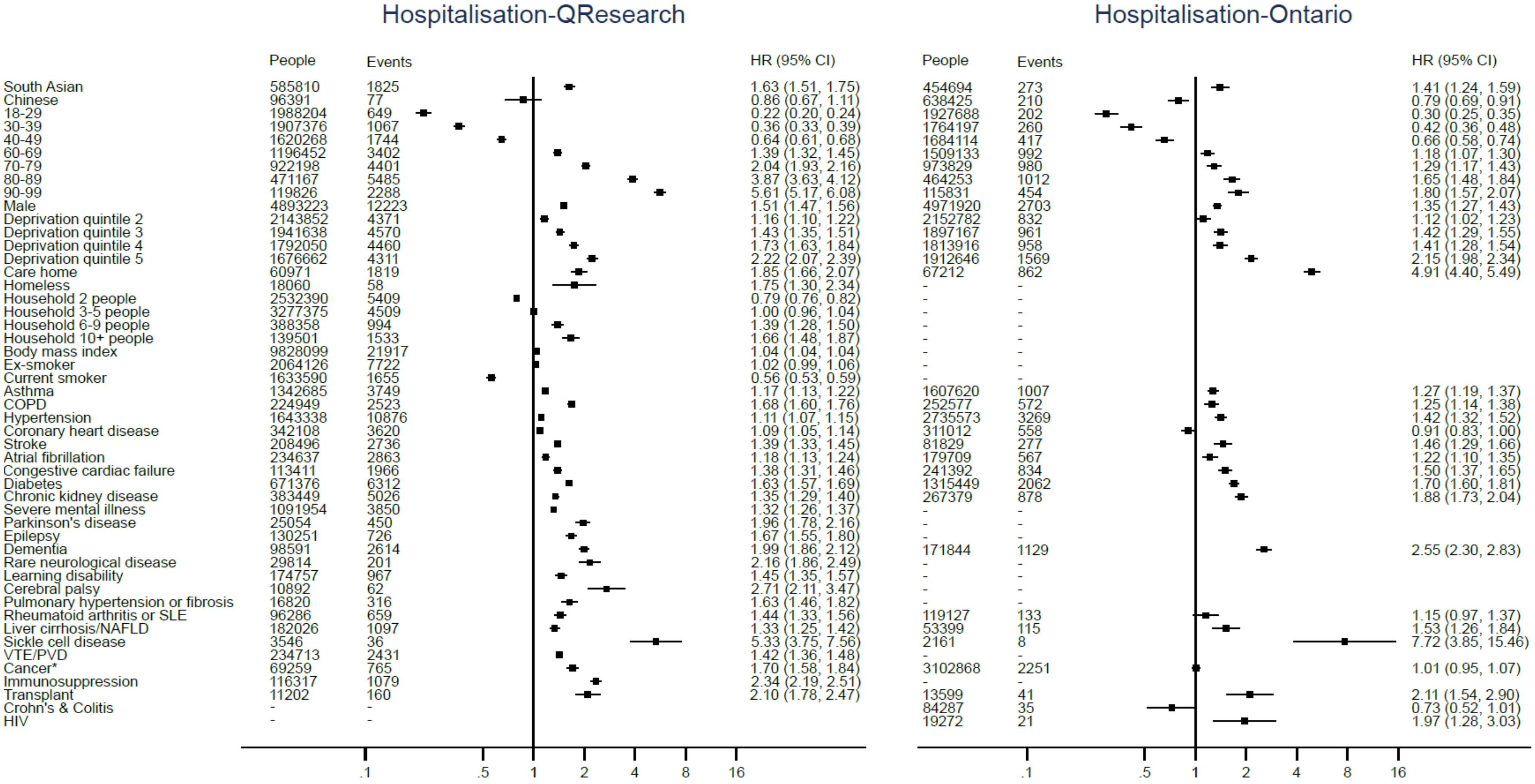

	QResearch People	QResearch Events	QResearch HR (95% CI)	Ontario People	Ontario Events	Ontario HR (95% CI)
South Asian	585810	1825	1.63 (1.51, 1.75)	454694	273	1.41 (1.24, 1.59)
Chinese	96391	77	0.86 (0.67, 1.11)	638425	210	0.79 (0.69, 0.91)
18-29	1988204	649	0.22 (0.20, 0.24)	1927688	202	0.30 (0.25, 0.35)
30-39	1907376	1067	0.36 (0.33, 0.39)	1764197	260	0.42 (0.36, 0.48)
40-49	1620268	1744	0.64 (0.61, 0.68)	1684114	417	0.66 (0.58, 0.74)
60-69	1196452	3402	1.39 (1.32, 1.45)	1509133	992	1.18 (1.07, 1.30)
70-79	922198	4401	2.04 (1.93, 2.16)	973829	980	1.29 (1.17, 1.43)
80-89	471167	5485	3.87 (3.63, 4.12)	464253	1012	1.65 (1.48, 1.84)
90-99	119826	2288	5.61 (5.17, 6.08)	115831	454	1.80 (1.57, 2.07)
Male	4893223	12223	1.51 (1.47, 1.56)	4971920	2703	1.35 (1.27, 1.43)
Deprivation quintile 2	2143852	4371	1.16 (1.10, 1.22)	2152782	832	1.12 (1.02, 1.23)
Deprivation quintile 3	1941638	4570	1.43 (1.35, 1.51)	1897167	961	1.42 (1.29, 1.55)
Deprivation quintile 4	1792050	4460	1.73 (1.63, 1.84)	1813916	958	1.41 (1.28, 1.54)
Deprivation quintile 5	1676662	4311	2.22 (2.07, 2.39)	1912646	1569	2.15 (1.98, 2.34)
Care home	60971	1819	1.85 (1.66, 2.07)	67212	862	4.91 (4.40, 5.49)
Homeless	18060	58	1.75 (1.30, 2.34)	-	-	
Household 2 people	2532390	5409	0.79 (0.76, 0.82)	-	-	
Household 3-5 people	3277375	4509	1.00 (0.96, 1.04)	-	-	
Household 6-9 people	388358	994	1.39 (1.28, 1.50)	-	-	
Household 10+ people	139501	1533	1.66 (1.48, 1.87)	-	-	
Body mass index	9828099	21917	1.04 (1.04, 1.04)	-	-	
Ex-smoker	2064126	7722	1.02 (0.99, 1.06)	-	-	
Current smoker	1633590	1655	0.56 (0.53, 0.59)	-	-	
Asthma	1342685	3749	1.17 (1.13, 1.22)	1607620	1007	1.27 (1.19, 1.37)
COPD	224949	2523	1.68 (1.60, 1.76)	252577	572	1.25 (1.14, 1.38)
Hypertension	1643338	10876	1.11 (1.07, 1.15)	2735573	3269	1.42 (1.32, 1.52)
Coronary heart disease	342108	3620	1.09 (1.05, 1.14)	311012	558	0.91 (0.83, 1.00)
Stroke	208496	2736	1.39 (1.33, 1.45)	81829	277	1.46 (1.29, 1.66)
Atrial fibrillation	234637	2863	1.18 (1.13, 1.24)	179709	567	1.22 (1.10, 1.35)
Congestive cardiac failure	113411	1966	1.38 (1.31, 1.46)	241392	834	1.50 (1.37, 1.65)
Diabetes	671376	6312	1.63 (1.57, 1.69)	1315449	2062	1.70 (1.60, 1.81)
Chronic kidney disease	383449	5026	1.35 (1.29, 1.40)	267379	878	1.88 (1.73, 2.04)
Severe mental illness	1091954	3850	1.32 (1.26, 1.37)	-	-	
Parkinson's disease	25054	450	1.96 (1.78, 2.16)	-	-	
Epilepsy	130251	726	1.67 (1.55, 1.80)	-	-	
Dementia	98591	2614	1.99 (1.86, 2.12)	171844	1129	2.55 (2.30, 2.83)
Rare neurological disease	29814	201	2.16 (1.86, 2.49)	-	-	
Learning disability	174757	967	1.45 (1.35, 1.57)	-	-	
Cerebral palsy	10892	62	2.71 (2.11, 3.47)	-	-	
Pulmonary hypertension or fibrosis	16820	316	1.63 (1.46, 1.82)	-	-	
Rheumatoid arthritis or SLE	96286	659	1.44 (1.33, 1.56)	119127	133	1.15 (0.97, 1.37)
Liver cirrhosis/NAFLD	182026	1097	1.33 (1.25, 1.42)	53399	115	1.53 (1.26, 1.84)
Sickle cell disease	3546	36	5.33 (3.75, 7.56)	2161	8	7.72 (3.85, 15.46)
VTE/PVD	234713	2431	1.42 (1.36, 1.48)	-	-	
Cancer*	69259	765	1.70 (1.58, 1.84)	3102868	2251	1.01 (0.95, 1.07)
Immunosuppression	116317	1079	2.34 (2.19, 2.51)	-	-	
Transplant	11202	160	2.10 (1.78, 2.47)	13599	41	2.11 (1.54, 2.90)
Crohn's & Colitis	-	-		84287	35	0.73 (0.52, 1.01)
HIV	-	-		19272	21	1.97 (1.28, 3.03)

Number of events and participants refer to complete-case data (Table 1, Table S1 and Table S2)

Rheumatoid arthritis alone in Ontario cohort. *Blood/respiratory cancer in QResearch, all cancer types in Ontario cohort.
The reference ethnic group is “general population”: people not South Asian and Chinese - Ontario: approximately 80% White; QResearch; White, Other Asian, Black African, Black Caribbean, and Other.
COPD – chronic obstructive pulmonary disease; SLE – systemic lupus erythematosus; NAFLD – Non-alcoholic fatty liver disease; VTE – venous thromboembolism; PVD – peripheral vascular disease; HIV – human immunodeficiency virus. Reference groups are: Ethnicity – White; Age – 50-59 years; Sex – Female; Deprivation – 1st quintile (least deprived); Residence – neither (QResearch), no long-term care resident (Ontario); Household size – 1 person; Smoking status – non-smoker. For body mass index (BMI, kg/m^2), hazard ratio is reported per 1-unit increase. For all comorbidities, reference is no disease (for chronic kidney disease [CKD] it is CKD stage 1-2)

Figure S3: Adjusted hazard ratios for COVID-19 ICU admission in QResearch and Ontario cohorts

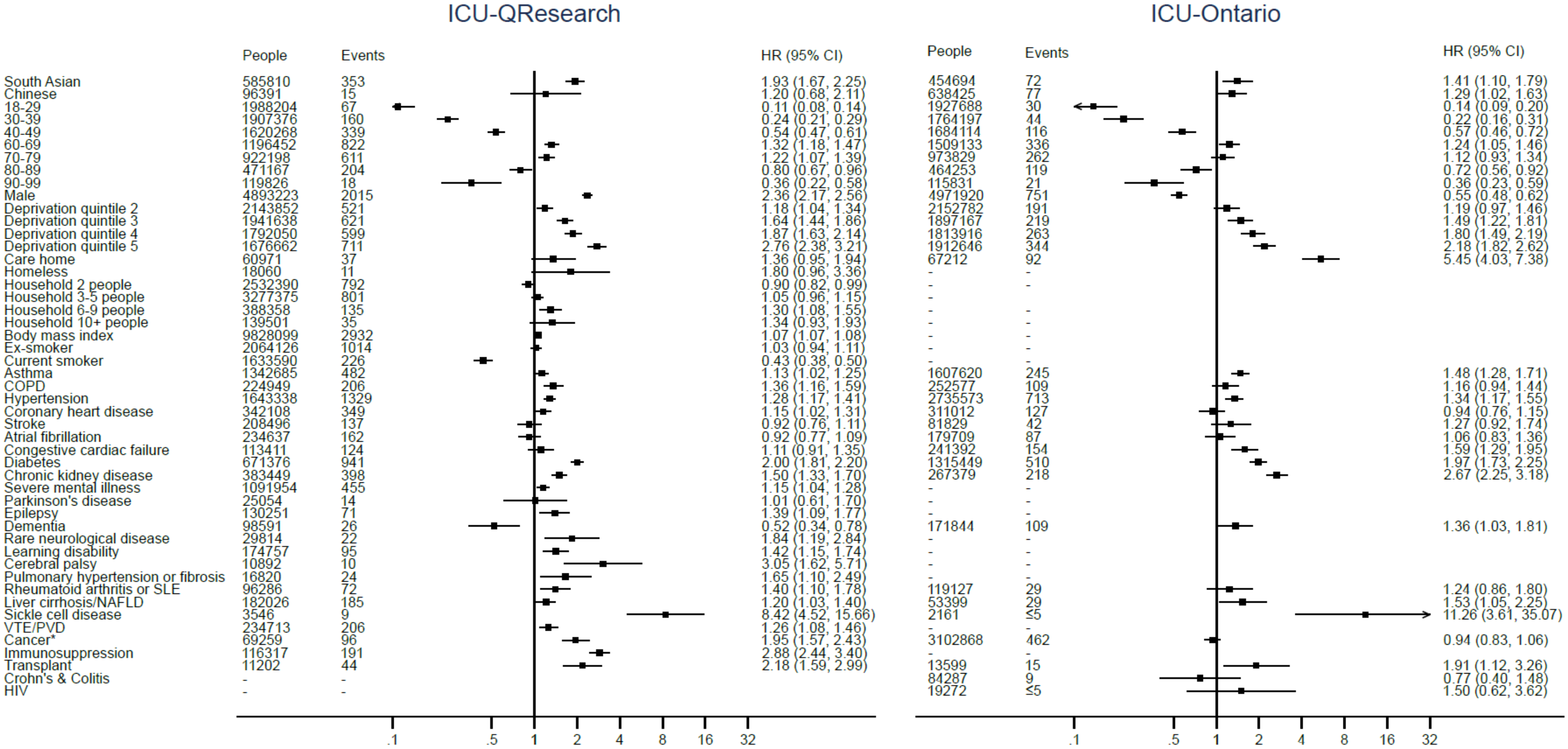

	ICU-QResearch People	ICU-QResearch Events	ICU-QResearch HR (95% CI)	ICU-Ontario People	ICU-Ontario Events	ICU-Ontario HR (95% CI)
South Asian	585810	353	1.93 (1.67, 2.25)	454694	72	1.41 (1.10, 1.79)
Chinese	96391	15	1.20 (0.68, 2.11)	638425	77	1.29 (1.02, 1.63)
18-29	1988204	67	0.11 (0.08, 0.14)	1927688	30	0.14 (0.09, 0.20)
30-39	1907376	160	0.24 (0.21, 0.29)	1764197	44	0.22 (0.16, 0.31)
40-49	1620268	339	0.54 (0.47, 0.61)	1684114	116	0.57 (0.46, 0.72)
60-69	1196452	822	1.32 (1.18, 1.47)	1509133	336	1.24 (1.05, 1.46)
70-79	922198	611	1.22 (1.07, 1.39)	973829	262	1.12 (0.93, 1.34)
80-89	471167	204	0.80 (0.67, 0.96)	464253	119	0.72 (0.56, 0.92)
90-99	119826	18	0.36 (0.22, 0.58)	115831	21	0.36 (0.23, 0.59)
Male	4893223	2015	2.36 (2.17, 2.56)	4971920	751	0.55 (0.48, 0.62)
Deprivation quintile 2	2143852	521	1.18 (1.04, 1.34)	2152782	191	1.19 (0.97, 1.46)
Deprivation quintile 3	1941638	621	1.64 (1.44, 1.86)	1897167	219	1.49 (1.22, 1.81)
Deprivation quintile 4	1792050	599	1.87 (1.63, 2.14)	1813916	263	1.80 (1.49, 2.19)
Deprivation quintile 5	1676662	711	2.76 (2.38, 3.21)	1912646	344	2.18 (1.82, 2.62)
Care home	60971	37	1.36 (0.95, 1.94)	67212	92	5.45 (4.03, 7.38)
Homeless	18060	11	1.80 (0.96, 3.36)	-	-	
Household 2 people	2532390	792	0.90 (0.82, 0.99)	-	-	
Household 3-5 people	3277375	801	1.05 (0.96, 1.15)	-		
Household 6-9 people	388358	135	1.30 (1.08, 1.55)	-	-	
Household 10+ people	139501	35	1.34 (0.93, 1.93)	-	-	
Body mass index	9828099	2932	1.07 (1.07, 1.08)	-	-	
Ex-smoker	2064126	1014	1.03 (0.94, 1.11)	-	-	
Current smoker	1633590	226	0.43 (0.38, 0.50)	-	-	
Asthma	1342685	482	1.13 (1.02, 1.25)	1607620	245	1.48 (1.28, 1.71)
COPD	224949	206	1.36 (1.16, 1.59)	252577	109	1.16 (0.94, 1.44)
Hypertension	1643338	1329	1.28 (1.17, 1.41)	2735573	713	1.34 (1.17, 1.55)
Coronary heart disease	342108	349	1.15 (1.02, 1.31)	311012	127	0.94 (0.76, 1.15)
Stroke	208496	137	0.92 (0.76, 1.11)	81829	42	1.27 (0.92, 1.74)
Atrial fibrillation	234637	162	0.92 (0.77, 1.09)	179709	87	1.06 (0.83, 1.36)
Congestive cardiac failure	113411	124	1.11 (0.91, 1.35)	241392	154	1.59 (1.29, 1.95)
Diabetes	671376	941	2.00 (1.81, 2.20)	1315449	510	1.97 (1.73, 2.25)
Chronic kidney disease	383449	398	1.50 (1.33, 1.70)	267379	218	2.67 (2.25, 3.18)
Severe mental illness	1091954	455	1.15 (1.04, 1.28)	-	-	
Parkinson's disease	25054	14	1.01 (0.61, 1.70)	-	-	
Epilepsy	130251	71	1.39 (1.09, 1.77)	-	-	
Dementia	98591	26	0.52 (0.34, 0.78)	171844	109	1.36 (1.03, 1.81)
Rare neurological disease	29814	22	1.84 (1.19, 2.84)	-	-	
Learning disability	174757	95	1.42 (1.15, 1.74)	-	-	
Cerebral palsy	10892	10	3.05 (1.62, 5.71)	-	-	
Pulmonary hypertension or fibrosis	16820	24	1.65 (1.10, 2.49)	-	-	
Rheumatoid arthritis or SLE	96286	72	1.40 (1.10, 1.78)	119127	29	1.24 (0.86, 1.80)
Liver cirrhosis/NAFLD	182026	185	1.20 (1.03, 1.40)	53399	29	1.53 (1.05, 2.25)
Sickle cell disease	3546	9	8.42 (4.52, 15.66)	2161	≤5	11.26 (3.61, 35.07)
VTE/PVD	234713	206	1.26 (1.08, 1.46)	-	-	
Cancer*	69259	96	1.95 (1.57, 2.43)	3102868	462	0.94 (0.83, 1.06)
Immunosuppression	116317	191	2.88 (2.44, 3.40)	-	-	
Transplant	11202	44	2.18 (1.59, 2.99)	13599	15	1.91 (1.12, 3.26)
Crohn's & Colitis	-	-		84287	9	0.77 (0.40, 1.48)
HIV	-	-		19272	≤5	1.50 (0.62, 3.62)

Number of events and participants refer to complete-case data (Table 1, Table S1 and Table S2)

Rheumatoid arthritis alone in Ontario cohort. *Blood/respiratory cancer in QResearch, all cancer types in Ontario cohort.
The reference ethnic group is "general population": people not South Asian and Chinese - Ontario: approximately 80% White; QResearch; White, Other Asian, Black African, Black Caribbean, and Other.
COPD – chronic obstructive pulmonary disease; SLE – systemic lupus erythematosus; NAFLD – Non-alcoholic fatty liver disease; VTE – venous thromboembolism; PVD – peripheral vascular disease; HIV – human immunodeficiency virus. Reference groups are: Ethnicity – White; Age – 50-59 years; Sex – Female; Deprivation – 1st quintile (least deprived); Residence – neither (QResearch), no long-term care resident (Ontario); Household size – 1 person; Smoking status – non-smoker. For body mass index (BMI, kg/m^2), hazard ratio is reported per 1-unit increase. For all comorbidities, reference is no disease (for chronic kidney disease [CKD] it is CKD stage 1-2).

Figure S4: Comparison between multiple imputed and complete case results (QResearch cohort)

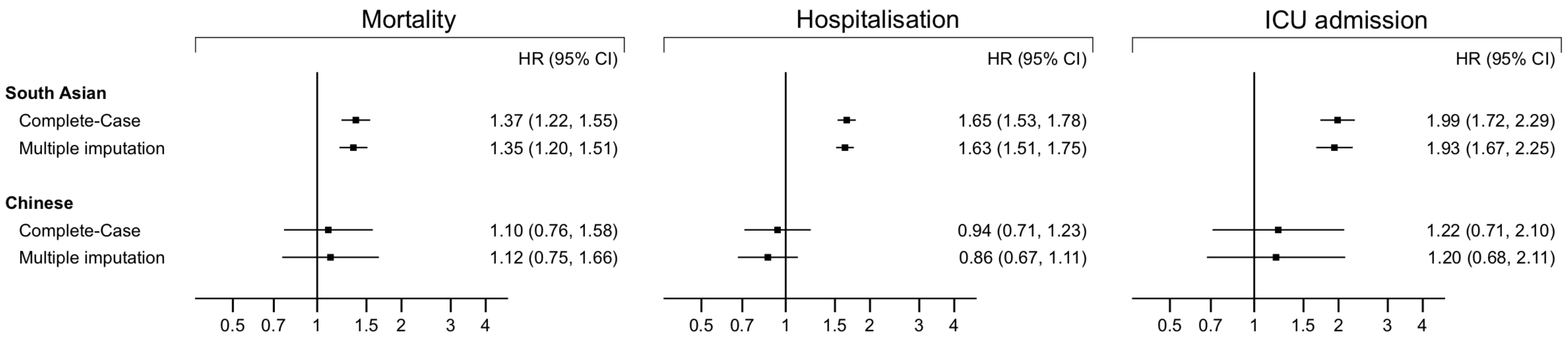

Number of events and participants are shown in Figure S1-S3 for complete case and Figure 1 following multiple imputation.

Figure S5: Time-varying hazard ratios of COVID-19 death and hospitalisation by ethnicity in the QResearch cohort

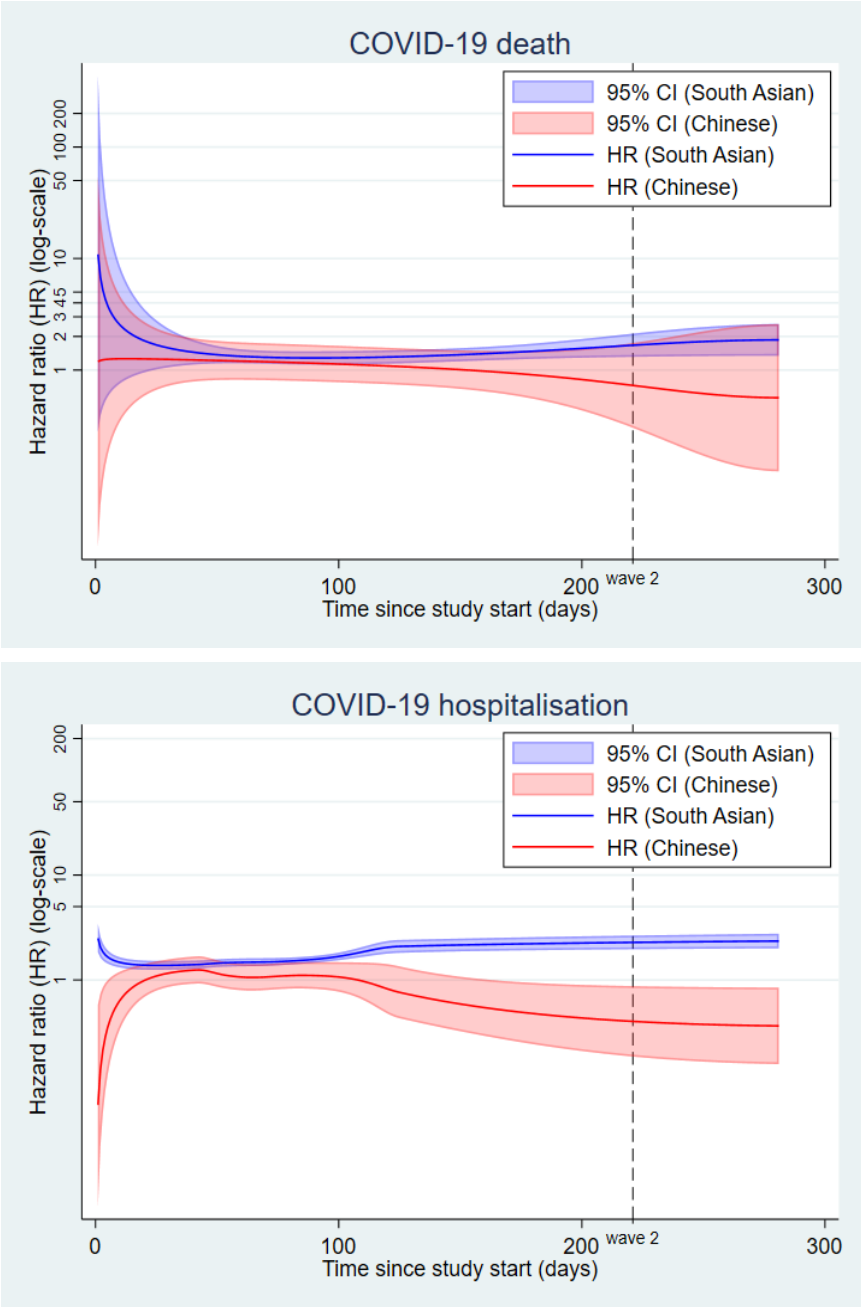

Areas indicate 95% CI. Dotted line represents the beginning of wave 2; wave 1: 24 January 2020 to 31 August 2020; wave 2: 1 September 2020 to 31 October 2020. Estimates from complete-case analysis.

Figure S6: COVID-19 related outcomes across deprivation status

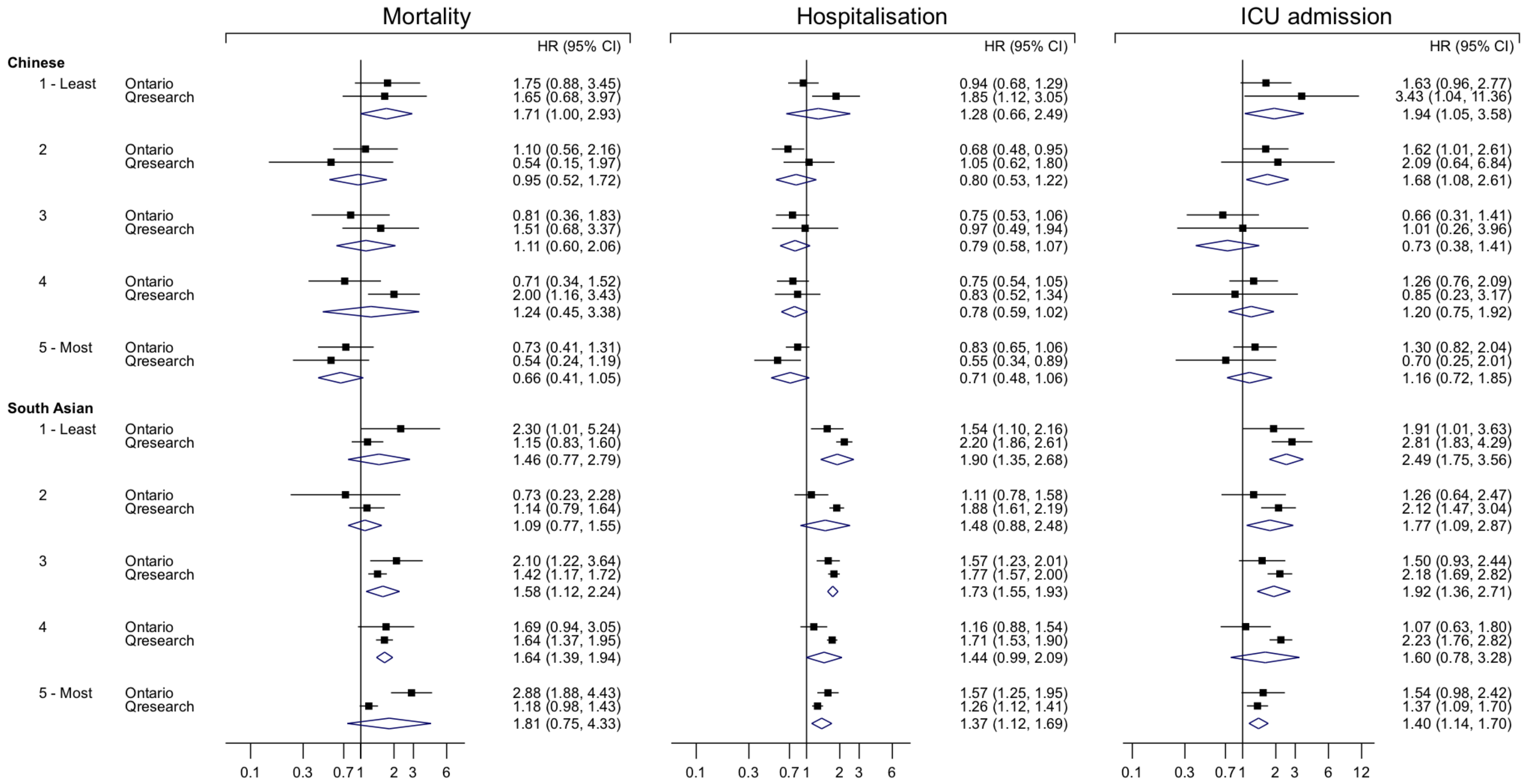

Hazard Ratios (HR) for each ethnic group are reported compared to the “General population” group

Log-negative-log plots – assessment of proportional hazards assumption

England (QResearch)

COVID-19 death

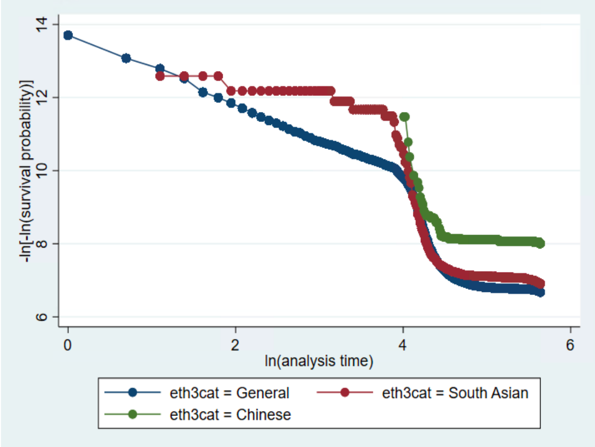

COVID-19 hospitalisation

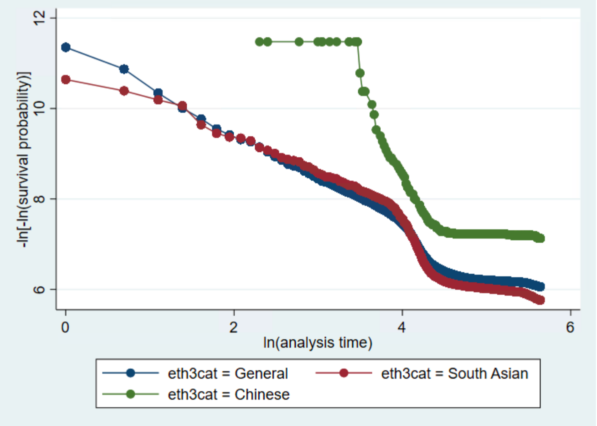

COVID-19 ICU admission

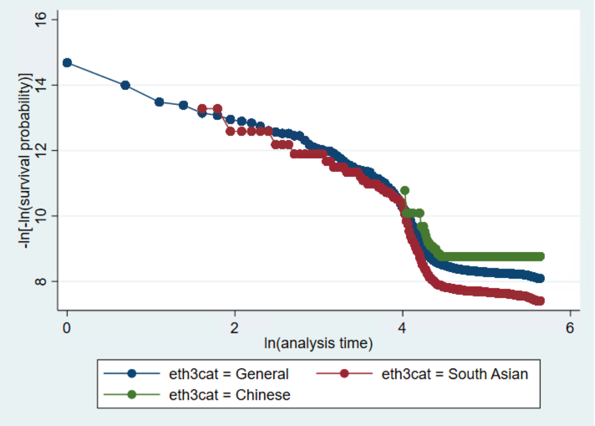

Ontario

COVID-19 death

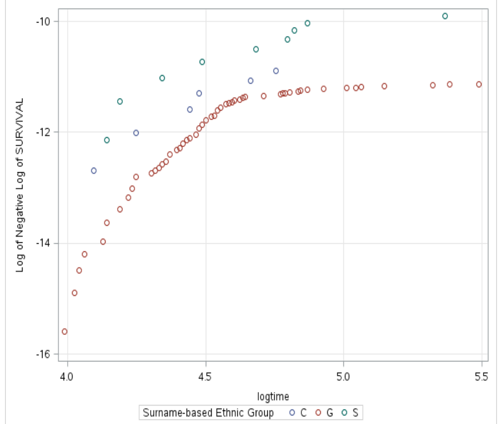

COVID-19 hospitalisation

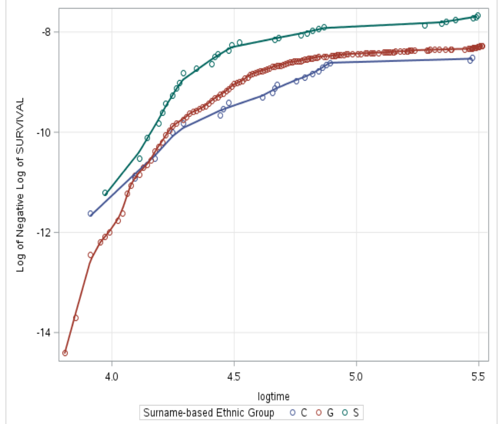

COVID-19 ICU admission

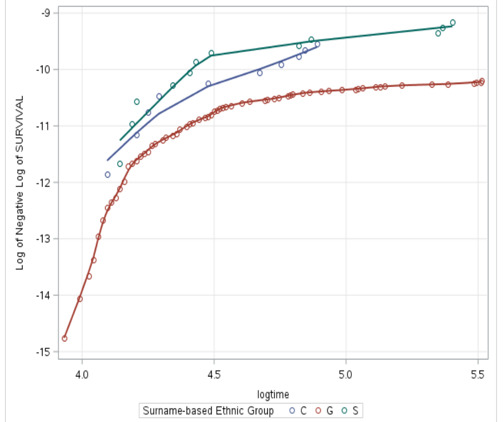

C = Chinese
G = General population
S = South Asian

The RECORD statement – checklist of items, extended from the STROBE statement that should be reported in observational studies using routinely collected health data.

	Item No.	STROBE items	Location in manuscript where items are reported	RECORD items	Location in manuscript where items are reported
**Title and abstract**					
	1	(a) Indicate the study's design with a commonly used term in the title or the abstract (b) Provide in the abstract an informative and balanced summary of what was done and what was found	Pages 1-2	RECORD 1.1: The type of data used should be specified in the title or abstract. When possible, the name of the databases used should be included.<br><br>RECORD 1.2: If applicable, the geographic region and timeframe within which the study took place should be reported in the title or abstract.<br><br>RECORD 1.3: If linkage between databases was conducted for the study, this should be clearly stated in the title or abstract.	Page 1-2
**Introduction**					
Background rationale	2	Explain the scientific background and rationale for the investigation being reported	Page 3		
Objectives	3	State specific objectives, including any prespecified hypotheses	Page 3		
**Methods**					
Study Design	4	Present key elements of study design early in the paper	Page 4		
Setting	5	Describe the setting, locations, and relevant dates, including periods of recruitment, exposure, follow-up, and data collection	Page 4, Suppl. Material		
Participants	6	*(a) Cohort study* - Give the eligibility criteria, and the sources and methods of selection of participants. Describe methods of follow-up	Page 4, Suppl. Material	RECORD 6.1: The methods of study population selection (such as codes or algorithms used to identify subjects) should be listed in detail. If this is not possible, an explanation should be provided.<br><br>RECORD 6.2: Any validation studies of the codes or algorithms used to select the population should be referenced. If	Page 4, Suppl. Material

				validation was conducted for this study and not published elsewhere, detailed methods and results should be provided.<br><br>RECORD 6.3: If the study involved linkage of databases, consider use of a flow diagram or other graphical display to demonstrate the data linkage process, including the number of individuals with linked data at each stage.	
Variables	7	Clearly define all outcomes, exposures, predictors, potential confounders, and effect modifiers. Give diagnostic criteria, if applicable.	Page 4, Suppl. Material	RECORD 7.1: A complete list of codes and algorithms used to classify exposures, outcomes, confounders, and effect modifiers should be provided. If these cannot be reported, an explanation should be provided.	Page 4, Suppl. Material
Data sources/ measurement	8	For each variable of interest, give sources of data and details of methods of assessment (measurement). Describe comparability of assessment methods if there is more than one group	Pages 4, Suppl. Material		
Bias	9	Describe any efforts to address potential sources of bias	Pages 4		
Study size	10	Explain how the study size was arrived at	Page 4-6		
Quantitative variables	11	Explain how quantitative variables were handled in the analyses. If applicable, describe which groupings were chosen, and why	Page 4, 5, Suppl. Material		
Statistical methods	12	(a) Describe all statistical methods, including those used to control for confounding<br>(b) Describe any methods used to examine subgroups and interactions<br>(c) Explain how missing data were addressed<br>(d) *Cohort study* - If applicable, explain how loss to follow-up was addressed<br>(e) Describe any sensitivity analyses	Pages 5, 6		
Data access and cleaning methods		..		RECORD 12.1: Authors should describe the extent to which the investigators had access to the database population used to create the study population.	Pages 4-6, 12

				RECORD 12.2: Authors should provide information on the data cleaning methods used in the study.	
Linkage				RECORD 12.3: State whether the study included person-level, institutional-level, or other data linkage across two or more databases. The methods of linkage and methods of linkage quality evaluation should be provided.	Page 5, Suppl. Material
**Results**					
Participants	13	(a) Report the numbers of individuals at each stage of the study (*e.g.*, numbers potentially eligible, examined for eligibility, confirmed eligible, included in the study, completing follow-up, and analysed)<br>(b) Give reasons for non-participation at each stage.<br>(c) Consider use of a flow diagram	Page 7	RECORD 13.1: Describe in detail the selection of the persons included in the study (*i.e.*, study population selection) including filtering based on data quality, data availability and linkage. The selection of included persons can be described in the text and/or by means of the study flow diagram.	Page 7
Descriptive data	14	(a) Give characteristics of study participants (*e.g.*, demographic, clinical, social) and information on exposures and potential confounders<br>(b) Indicate the number of participants with missing data for each variable of interest<br>(c) *Cohort study* - summarise follow-up time (*e.g.*, average and total amount)	Page 7		
Outcome data	15	*Cohort study* - Report numbers of outcome events or summary measures over time	Page 7		
Main results	16	(a) Give unadjusted estimates and, if applicable, confounder-adjusted estimates and their precision (e.g., 95% confidence interval). Make clear which confounders were adjusted for and why they were included<br>(b) Report category boundaries when continuous variables were categorized<br>(c) If relevant, consider translating estimates of relative risk into absolute risk for a meaningful time period	Page 7		
Other analyses	17	Report other analyses done—e.g., analyses of subgroups and interactions, and sensitivity analyses	Page 8		
**Discussion**					

Key results	18	Summarise key results with reference to study objectives	Page 9		
Limitations	19	Discuss limitations of the study, taking into account sources of potential bias or imprecision. Discuss both direction and magnitude of any potential bias	Page 10	RECORD 19.1: Discuss the implications of using data that were not created or collected to answer the specific research question(s). Include discussion of misclassification bias, unmeasured confounding, missing data, and changing eligibility over time, as they pertain to the study being reported.	Page 9-11
Interpretation	20	Give a cautious overall interpretation of results considering objectives, limitations, multiplicity of analyses, results from similar studies, and other relevant evidence	Pages 10-11		
Generalisability	21	Discuss the generalisability (external validity) of the study results	Pages 10-11		
**Other Information**					
Funding	22	Give the source of funding and the role of the funders for the present study and, if applicable, for the original study on which the present article is based	Page 11		
Accessibility of protocol, raw data, and programming code		..	Page 12	RECORD 22.1: Authors should provide information on how to access any supplemental information such as the study protocol, raw data, or programming code.	Page 12, Suppl. Material

Benchimol EI, Smeeth L, Guttmann A, Harron K, Moher D, Petersen I, Sørensen HT, von Elm E, Langan SM, the RECORD Working Committee. The REporting of studies Conducted using Observational Routinely-collected health Data (RECORD) Statement. PLoS Med. 2015 Oct 6;12(10):e1001885. doi: 10.1371/journal.pmed.1001885.

Checklist is protected under Creative Commons Attribution (CC BY) license.

Page numbers refer to the original, word document submission.
